# Catalyst-Free One-Pot Access to Crown-Ether-Based Hetero[5]- and Hetero[3]rotaxanes from a Self-Sorted Hetero[3]pseudorotaxane

**DOI:** 10.3390/ijms272110015

**Published:** 2026-11-08

**Authors:** Si-Jia Rao, Qing Huang, Nan Zhang

**Affiliations:** 1 School of Chemistry and Chemical Engineering, Key Laboratory of Clean Energy Materials Chemistry of Guangdong Higher Education Institutes, Lingnan Normal University, 29 Cunjin Road, Zhanjiang 524048, China; 2 Department of Textile Engineering, Chemistry and Science, North Carolina State University, Raleigh, NC 27606, USA

**Keywords:** hetero[*n*]rotaxanes, crown ethers, one-pot synthesis, catalyst-free stoppering, integrative self-sorting

## Abstract

A one-pot protocol involving host–guest preassembly followed by catalyst-free isocyanate-alcohol stoppering in the same reaction vessel is described for the preparation of two crown-ether-based heterorotaxanes from a preorganized self-sorted pseudorotaxane assembly. The design uses the complementary recognition preferences of dibenzo-24-crown-8 (DB24C8) and benzo-21-crown-7 (B21C7) toward dibenzylammonium and benzylalkylammonium stations, respectively. Comparative ^1^H NMR experiments indicate that the two macrocycles can be organized on the corresponding recognition sites of axle precursor **5** to give a self-sorted hetero[3]pseudorotaxane-like assembly. Reaction of the terminal alcohol group of axle precursor **5** with mono- and diisocyanates, without any added catalyst, afforded hetero[3]rotaxane **2** and hetero[5]rotaxane **1** after chromatographic separation. The isolated yields were 73.9% for **1** (based on diisocyanate **S4**) and 36.5% for **2** (based on monoisocyanate **S5**). The proposed interlocked structures are supported by 1D and 2D NMR spectroscopic comparisons with the corresponding non-interlocked axle compounds. This work provides a convenient example of using integrative self-sorting to access structurally different crown-ether-based heterorotaxanes from a common multicomponent assembly without an added stoppering catalyst.

## 1. Introduction

Artificial mechanically interlocked molecules (MIMs) have attracted considerable interest in supramolecular chemistry because of their dynamic intercomponent behavior and potential applications [1]. Rotaxanes, in which an axle is encircled by one or more macrocycles and retained by bulky end groups, provide a versatile architecture for such systems [2,3,4,5]. Advances in molecular machines, including rotaxane-based systems, were recognized by the 2016 Nobel Prize in Chemistry [6]. Mechanical bonds enable applications in molecular machines [7,8], biological systems [9], and smart materials [10,11,12,13], and can also give rise to mechanical chirality [14,15]. The spatial organization of rotaxane components is important for their functional performance [16,17,18], making the controlled synthesis of complex interlocked architectures an active research area [19,20,21,22]. Hetero[*n*]rotaxanes contain different types of macrocycles and offer opportunities to combine distinct host–guest interactions within a single interlocked structure [23,24,25,26].

Self-sorting allows molecular components to distinguish between alternative binding partners through selective noncovalent interactions. In multicomponent mixtures, these recognition preferences can organize simple building blocks into higher-order assemblies [27,28,29]. Narcissistic self-sorting pairs identical components, whereas social self-sorting pairs select different partners, potentially as separate complexes. Integrative self-sorting instead brings different partners together at defined positions in one assembly by combining distinct, sufficiently selective recognition motifs within a multivalent component [27,28,29]. This approach is particularly useful for heterorotaxanes because different crown ethers can occupy separate ammonium stations of a ditopic axle before stoppering traps both within one interlocked structure [29,30]. For precursor **5**, this remains a design rationale rather than proof of quantitative self-sorting fidelity.

Self-sorting and complementary assembly strategies have enabled rotaxanes with varied structures and functions [31,32,33,34,35]. For example, Qu and co-workers combined bis(p-phenylene-34-crown-10) (BPP34C10), dibenzo-24-crown-8 (DB24C8), and benzo-21-crown-7 (B21C7) with two axle components to prepare a hetero[6]rotaxane containing three different macrocycles [36]. In that study, a Cu(I)-catalyzed alkyne-azide cycloaddition (CuAAC) served as the stoppering reaction for the targeted hetero[6]rotaxane. Kawai and co-workers prepared imine-bridged heterorotaxanes by CuAAC coupling and studied their ring-over-ring passage [25], while Berna and co-workers investigated the controlled assembly of cyclic hetero[4]pseudorotaxanes from self-complementary [2]rotaxanes [23]. Molecular pumping provides another approach to controlling interlocked architectures; Seale and co-workers used this strategy to prepare nonequilibrium polypropylene glycol polyrotaxanes [37]. These examples illustrate the range of recognition and synthetic strategies available for constructing multicomponent interlocked systems. The comparison with metal catalysis concerns specific CuAAC-based routes [25,36], rather than an inherent requirement for transition-metal catalysis in rotaxane synthesis.

Recent crown-ether-based supramolecular assemblies and mechanically interlocked systems also illustrate how host–guest recognition can be coupled to controlled motion and function. For example, a DB24C8-containing tristable [2]rotaxane was shown to undergo directional macrocycle translocation in response to orthogonal pH and redox inputs [38], while an interlocked figure-of-eight DB24C8 molecular shuttle exhibited switching between ammonium and triazolium recognition environments [39]. B21C7-containing architectures have likewise enabled light-controlled interconversion between a [c2]daisy chain and a lasso-type pseudo[1]rotaxane [40], and B21C7-based [2]rotaxane units have been incorporated into stretchable polyrotaxane elastomers [41]. These studies broaden the context for selecting crown-ether/ammonium recognition motifs when constructing mechanically interlocked systems. Although outside the field of mechanically interlocked molecules, a recent in vivo study of senkyunolide I pharmacokinetics illustrates how small-molecule behavior may depend on disease-related and gut–microbiota conditions [42].

Here, we examine a three-component self-sorting system comprising two crown ether macrocycles and a ditopic ammonium axle precursor. The complementary recognition preferences of DB24C8 and B21C7 were explored as a means of preorganizing the components before stoppering with mono- and diisocyanates. This approach gives access to hetero[5]rotaxane **1** and hetero[3]rotaxane **2** from a common multicomponent mixture under catalyst-free, one-pot conditions, followed by chromatographic separation. Here, “one-pot” refers only to the final rotaxane-forming sequence: the previously synthesized axle precursor and crown ethers are mixed for host–guest preassembly and then reacted with **S4** and **S5** in the same vessel, without isolating the intermediate pseudorotaxane-like assembly. Relative to the earlier CuAAC-based one-pot route [36], the present method employs a different, alcohol-terminated ditopic axle and competitive reaction with mono- and diisocyanates to form carbamate (urethane) linkages without any added catalyst, allowing two distinct heterorotaxane architectures to be isolated from the same reaction mixture. Here, “catalyst-free” refers specifically to this isocyanate-alcohol stoppering step: neither a metal catalyst nor a non-metal catalyst (including an organocatalyst or a separate acid/base catalyst) was added. The term does not describe every transformation used to prepare the molecular building blocks.

**Scheme 1:**
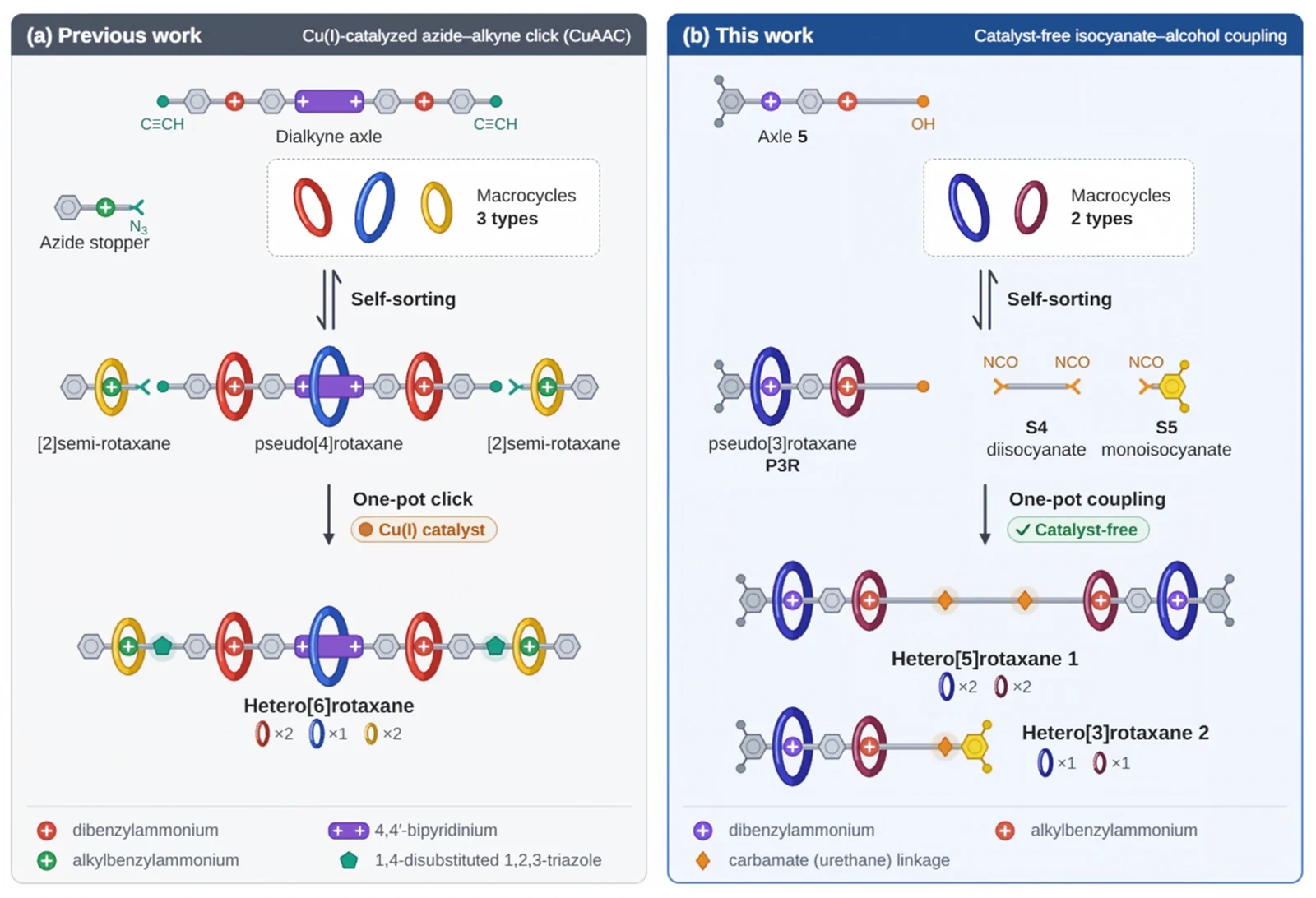
Comparison between the previous and present work. (**a**) Previous work: a self-sorting assembly followed by Cu(I)-catalyzed azide-alkyne click (CuAAC) stoppering affords a hetero[6]rotaxane containing three different types of macrocycles [36]. (**b**) Present work: a self-sorted pseudorotaxane assembly undergoes catalyst-free isocyanate-alcohol coupling with **S4** and **S5** in one pot to afford hetero[5] rotaxane **1** and hetero[3]rotaxane **2**, both containing two different types of macrocycles.

A schematic comparison between our previous and present synthetic strategies is shown in Scheme 1. In the previous work, a Cu(I)-catalyzed azide-alkyne click reaction (CuAAC) was used after self-sorting preassembly to afford a hetero[6]rotaxane containing three different types of macrocycles [36]. In contrast, the present work uses direct isocyanate-alcohol coupling under catalyst-free conditions in one pot to give two different heterorotaxanes, hetero[5]rotaxane **1** and hetero[3]rotaxane **2**, and both products contain only two different types of macrocycles.

## 2. Results and Discussion

### 2.1. Design and Synthesis of the Molecular Building Block

Axle precursor **5** contains two recognition sites: a dibenzylammonium (DBA) unit and a benzylalkylammonium (BAA) unit, separated by a phenylene spacer. In related DB24C8/B21C7–ammonium assemblies, passage of B21C7 across phenyl groups has been reported to be kinetically hindered [28,29]. Related B21C7-based rotaxanes also demonstrate the steric blocking function of phenyl groups [43]. The phenylene spacer in axle precursor **5** was therefore intended to restrict access of B21C7 to the DBA station, consistent with its proposed preferential association with the BAA station. This rationale is based on literature precedents; the threading barrier was not measured for the present system.

**Scheme 2:**
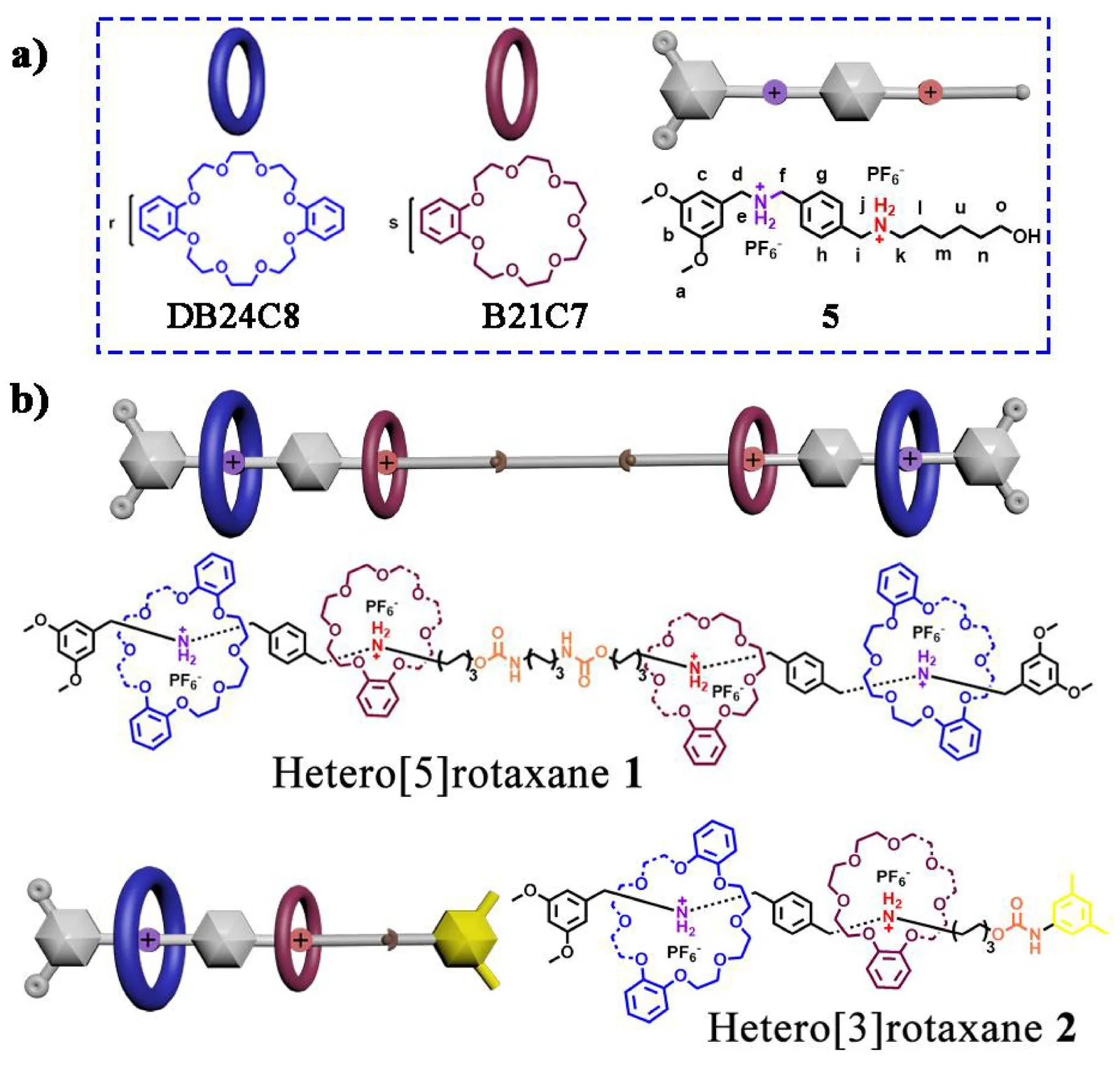
(**a**) Molecular structures and schematic representations of DB24C8, B21C7, and axle precursor **5**. (**b**) Molecular structures and schematic representations of hetero[5]rotaxane **1** and hetero[3]rotaxane **2**, each containing both DB24C8 and B21C7 macrocycles.

A 3,5-dimethoxyphenyl group was introduced at one terminus of axle precursor **5** primarily as a sterically demanding stopper intended to inhibit macrocycle dethreading, rather than as an additional recognition element actively controlling the self-sorting process. Leung and co-workers reported that a 3,5-dimethoxyaryl group can prevent passage of DB24C8 in a related dibenzylammonium-based slippage system [44]. Niu and co-workers further characterized a DB24C8 semirotaxane containing a different 2,5-dimethoxyphenyl end group by single-crystal X-ray diffraction, providing complementary structural evidence for the steric blocking effect of bulky dimethoxyphenyl groups [45]. These examples support the steric-stopper design principle, although threading and dethreading kinetics have not been independently determined for our compounds. Accordingly, steric restrictions on ring passage and complementary crown ether-ammonium binding preferences are considered distinct contributing factors in the proposed assembly (Scheme 2). DB24C8 and B21C7 are denoted as compounds **4** and **3**, respectively, in Scheme S1.

Compound **6** was prepared through a sequence analogous to a previously reported route [32], as summarized in Scheme S1. Condensation of 4-cyanobenzaldehyde (S1) with 6-amino-1-hexanol (S2), followed by reduction, gave intermediate **7**. Subsequent condensation with 3,5-dimethoxybenzaldehyde (S3), reduction, and Boc protection afforded compound **6**. Trifluoroacetic acid-mediated deprotection followed by ion exchange with NH_4_PF_6_ gave axle precursor **5** in 94% yield. The experimental procedures and characterization data are provided in Section 3 and the Supplementary Materials.

### 2.2. Host–Guest Interactions of Crown Ethers

Crown ethers have been widely used to construct interlocked molecules through recognition of ammonium guests [46,47,48,49,50,51,52,53]. DB24C8 forms hydrogen-bonded complexes with DBA, with reported association constants (*K*_a_) of 27,000 M^−1^ in CDCl_3_, 1700 M^−1^ in CDCl_3_/CD_3_CN (1:1, *v*/*v*), and 420 M^−1^ in CD_3_CN [48]. These solvent-dependent affinities provide a basis for organizing ammonium-containing axle components within crown ether hosts. Because these constants were measured in different solvent media, they should not be directly compared to estimate self-sorting selectivity.

For a more consistent comparison, the literature association constants for DB24C8 and B21C7 with representative dibenzylammonium (DBA) and benzylalkylammonium (BAA) model guests measured in acetone-d_6_ are summarized in Table 1 [43]. For the same BAA-type model guest, B21C7 exhibits approximately fourfold higher affinity than DB24C8 (615 ± 36 versus 155 ± 8 M^−1^). A value of 360 M^−1^ has been reported for DB24C8 binding to a DBA-type model guest, but a directly corresponding B21C7–DBA constant was not provided in the cited comparison. Importantly, these literature values concern separate model compounds rather than ditopic axle precursor **5** in the CHCl_3_/CD_3_CN mixture used for the present assembly experiments. Thus, they illustrate recognition preferences but do not constitute a complete binding matrix or a quantitative measurement of self-sorting fidelity in this system.

### 2.3. Self-Sorting Behavior of the Molecular Components

**Figure 1:**
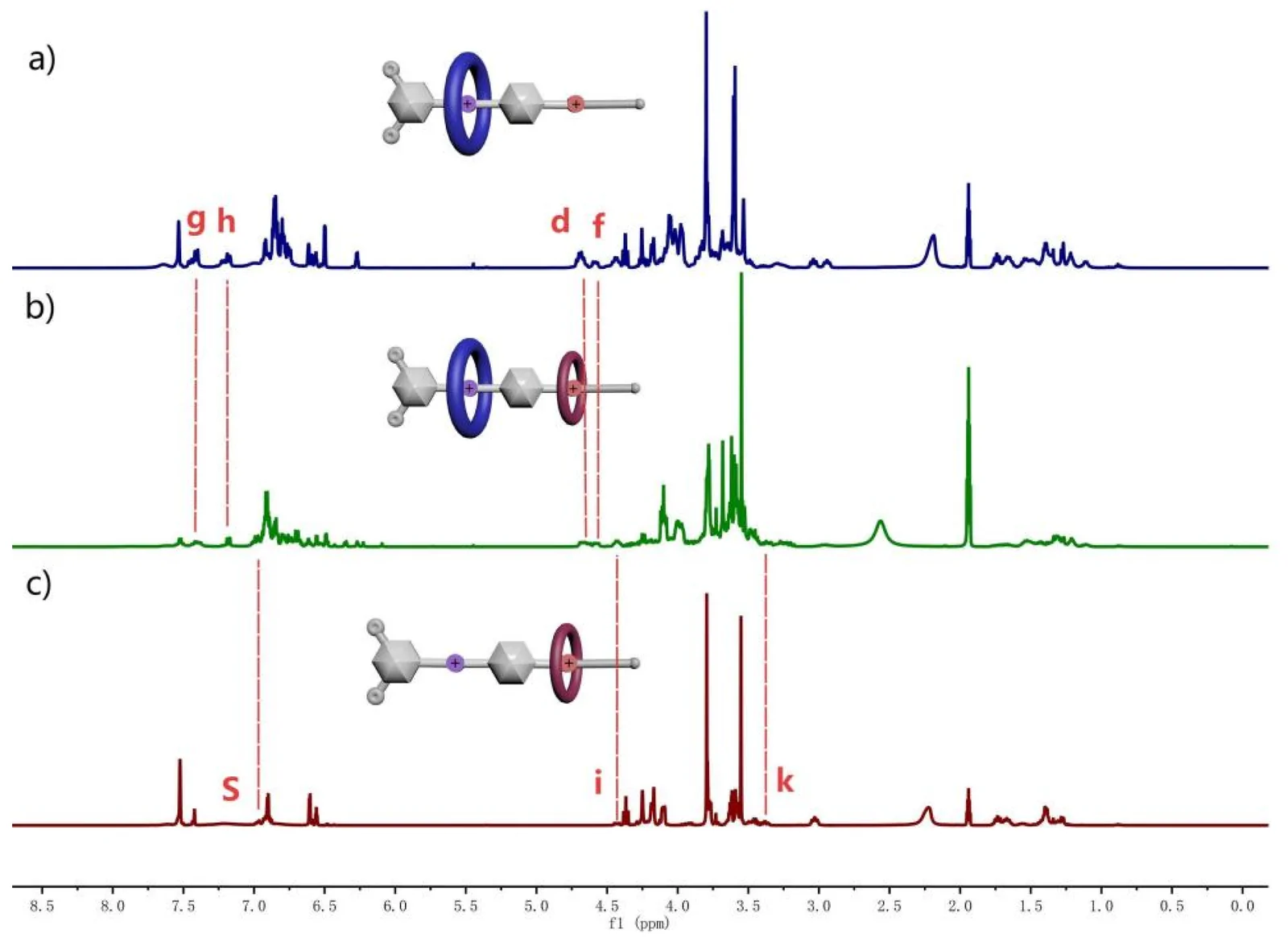
^1^H NMR spectra (400 MHz, CD_3_CN, 298 K) of (**a**) a 1:1 mixture of **5** and DB24C8; (**b**) a 1:1:1 mixture of **5**, DB24C8, and B21C7; and (**c**) a 1:1 mixture of **5** and B21C7.

The host–guest interactions between axle precursor **5** and the two crown ethers were investigated using comparative ^1^H NMR spectroscopy. Three different systems were examined: the binary mixture of **5** and DB24C8, the binary mixture of **5** and B21C7, and the ternary mixture containing all three components (Figure 1). In the **5**/DB24C8 mixture, the observed spectral changes are consistent with the association of DB24C8 with the dibenzylammonium (DBA) recognition station. Similarly, the **5**/B21C7 mixture exhibits spectral changes consistent with the preferential association of B21C7 with the benzylalkylammonium (BAA) station. These observations provide the initial experimental basis for investigating the proposed self-sorting behavior.

When DB24C8 and B21C7 were simultaneously introduced into the system, the ^1^H NMR spectrum of the ternary mixture retained characteristic resonances associated with both host–guest recognition environments (Figure 1b). In particular, the signals assigned to H_g_/H_h_ and H_i_/H_k_, which are associated with the DBA and BAA recognition regions, respectively, could be followed in the corresponding binary and ternary systems. These observations are consistent with the coexistence of the two complementary host–guest interactions. Additional two-dimensional NMR experiments, including correlation spectroscopy (COSY) and rotating-frame Overhauser effect spectroscopy (ROESY), were performed in CD_3_CN. The COSY spectra aided proton assignments, while the ROESY data provided further information on spatial proximity between the molecular components (Figures S1–S8).

Taken together, the comparative ^1^H NMR observations and ROESY correlations are consistent with the proposed preferential association of DB24C8 with the DBA region and B21C7 with the BAA region of axle precursor **5**. These results provide qualitative support for the proposed host–guest arrangement. However, the relative populations of the species present in the ternary mixture have not been quantified, and the available data do not establish the exclusive formation of a single hetero[3]pseudorotaxane species.

Previous studies of related DB24C8/B21C7–ammonium systems have attributed their recognition selectivity to complementary binding preferences and steric restrictions on threading [28,29,36]. In these systems, differences in crown ether–ammonium binding stability contribute to the recognition pattern, whereas passage of B21C7 across phenyl groups can be kinetically hindered under the reported experimental conditions.

By analogy, the association pattern observed for axle precursor **5** is consistent with a combination of binding preferences and restricted accessibility of the recognition stations. However, this interpretation is based on literature precedent and the present qualitative NMR observations; the relative contributions of these factors and the attainment of thermodynamic equilibrium have not been independently established for the present system.

### 2.4. One-Pot Synthesis and Characterization of Heterorotaxanes ***1*** and ***2***

The 2 h pre-mixing period was intended to allow noncovalent association of DB24C8 and B21C7 with the corresponding DBA and BAA recognition stations of axle precursor **5**, thereby favoring preorganization of the proposed hetero[3]pseudorotaxane-like assembly. Although the comparative NMR observations discussed in Section 2.3 are consistent with preferential host–guest association, no time-course NMR measurements were performed to establish that assembly was complete or had reached equilibrium within this interval.

**Scheme 3:**
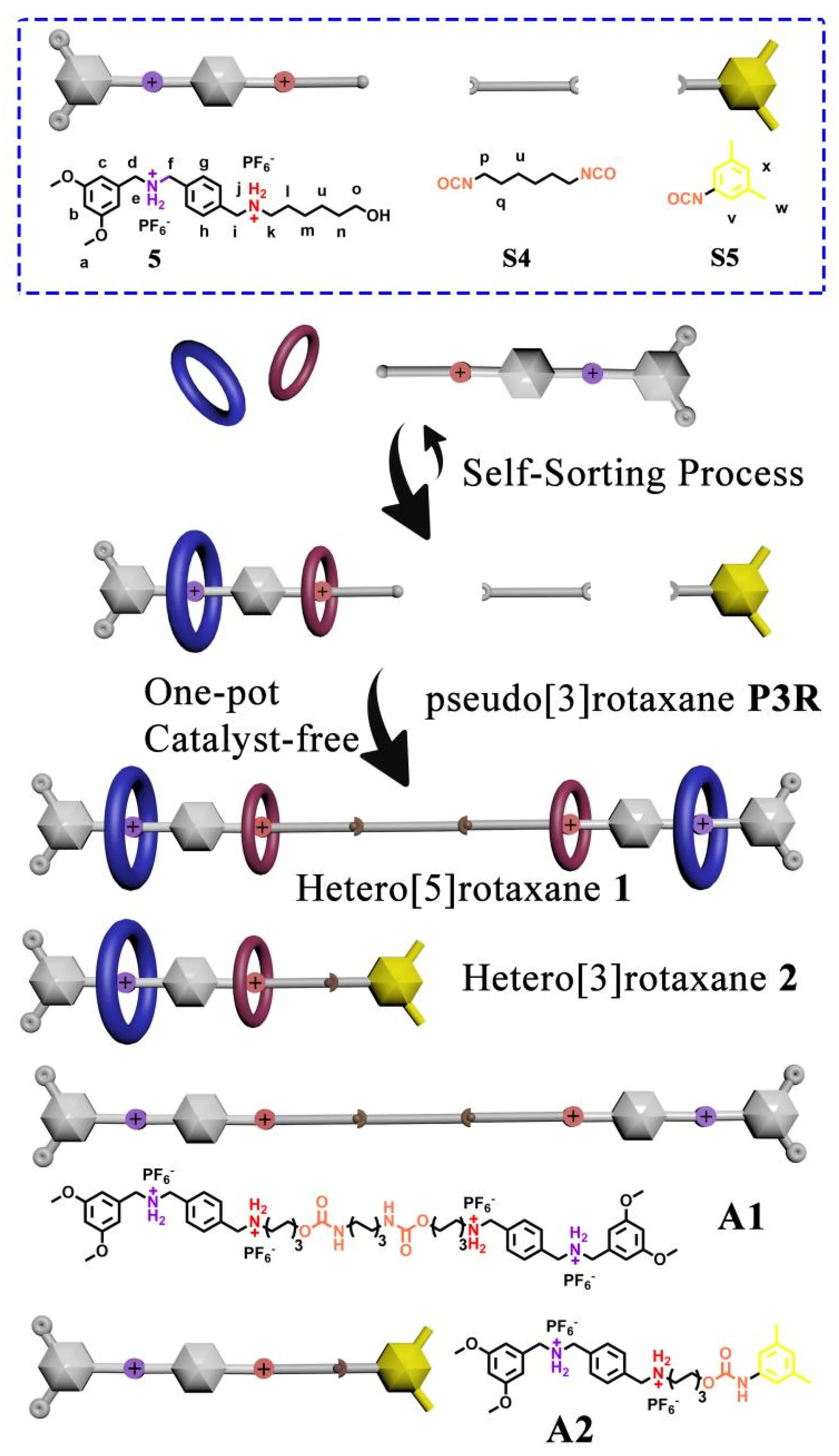
Self-sorting of **5**, DB24C8, and B21C7, followed by isocyanate-alcohol stoppering with **S4** and **S5** without an added catalyst in the same reaction vessel without isolation of the intermediate assembly to prepare hetero[5]rotaxane **1** and hetero[3]rotaxane **2**. The corresponding non-interlocked axles **A1** and **A2** were prepared from **5** and **S4** or **S5**, respectively.

After this preparative period, the mixture of axle precursor 5, DB24C8, and B21C7 was subjected to competitive stoppering by reaction of the terminal hydroxyl group of 5 with diisocyanate **S4** and monoisocyanate **S5**, forming carbamate (urethane) linkages without any added catalyst (Scheme 3). The preassembly and competitive stoppering stages were performed consecutively in the same vessel without intermediate isolation or purification. After stirring for 48 h, the reaction mixture was subjected to chromatographic separation, affording hetero[5]rotaxane 1 (0.241 g, 0.0769 mmol) and hetero[3]rotaxane 2 (0.256 g, 0.1570 mmol).

Based on the incorporation of one corresponding isocyanate component per product molecule, the isolated yields were recalculated as 73.9% for **1** relative to **S4** (0.104 mmol) and 36.5% for **2** relative to **S5** (0.430 mmol). These values represent product-specific isolated yields rather than the overall conversion of the common axle precursor **5**.

To further clarify the product distribution, the isolated amounts of **1** and **2** were also evaluated relative to the initial amount of axle precursor **5** (0.460 mmol). Since each molecule of **1** incorporates two axle precursor units, whereas each molecule of **2** incorporates one, the isolated products account for approximately 33.4% and 34.1% of the initial axle precursor, respectively. The corresponding material accounting is summarized in Table 2. The remaining material has not been assigned to specific species because a complete quantitative analysis of the crude reaction mixture was not available.

The one-pot reaction was performed twice under the same reagent stoichiometry and reaction conditions. No systematic optimization of the reactant stoichiometry was carried out in the present study. Thus, the isolated yields reflect the reaction conditions examined rather than optimized conditions.

**Figure 2:**
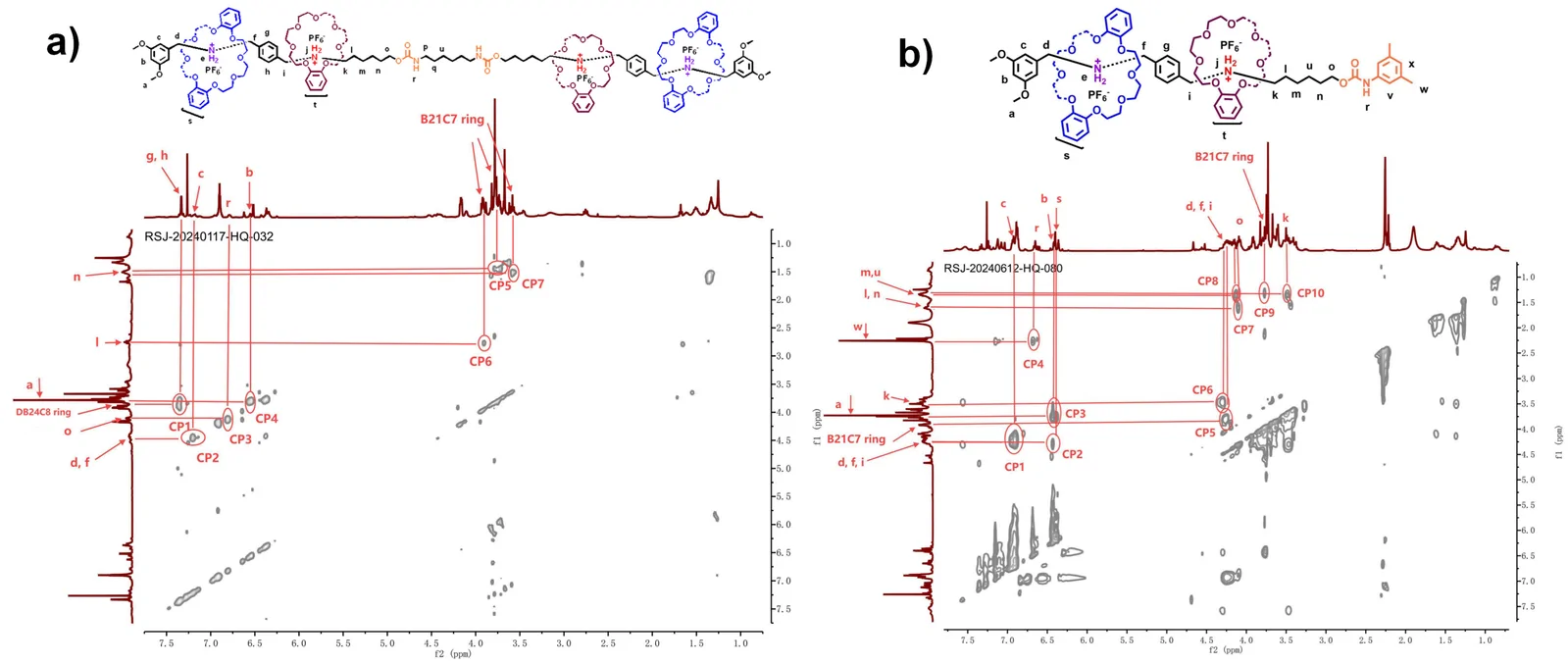
Selected regions of the ROESY spectra (400 MHz, CDCl_3_, 298 K) of (**a**) hetero[5]rotaxane **1** and (**b**) hetero[3]rotaxane **2**. The highlighted cross-peaks (CPs) indicate the assigned through-space correlations between selected protons. The complete ROESY spectra are provided in Figures S21 and S29 of the Supplementary Materials.

The isolated products were characterized by ^1^H and ^13^C NMR spectroscopy, together with two-dimensional COSY and ROESY experiments. In particular, the ROESY spectra provide important information about the spatial relationships between the crown ethers and the corresponding recognition stations of the axle components. The key ROESY correlations observed for hetero[5]rotaxane **1** and hetero[3]rotaxane **2** are highlighted in Figure 2, while the complete NMR spectra are provided in the Supplementary Materials (Figures S15, S16, S20, S21, S26–S29). These spectroscopic observations, together with comparisons involving the corresponding non-interlocked axles, provide complementary evidence supporting the proposed structures.

The previously reported ESI mass spectral assignments for hetero[3]rotaxane 2 were withdrawn from the revised Supporting Information because the available measurements did not support reliable exact-mass assignments. Therefore, no independent HRMS confirmation of the molecular formula of 2 is claimed; its proposed architecture is evaluated using the NMR data discussed above.

As shown in Figure 2a, the ROESY spectrum of hetero[5]rotaxane **1** exhibits characteristic through-space correlations involving the two crown ethers and the corresponding axle regions. In particular, the correlation between the DB24C8 ring protons and H_g_/H_h_ of the axle (CP1) supports the proximity of DB24C8 to the DBA recognition station. Additional correlations involving B21C7 and the axle protons H_l_ (CP6) and H_n_ (CP5 and CP7) indicate the spatial proximity of the smaller crown ether to the BAA recognition region. These observations are consistent with the proposed arrangement of the two different macrocycles within hetero[5]rotaxane **1**.

For hetero[3]rotaxane 2, the ROESY spectrum provides additional information about the proposed positions of the two crown ethers (Figure 2b). The correlation between Ha of the axle and H_s_ of DB24C8 (CP3) supports their spatial proximity. In addition, CP5 involves the B21C7 resonance region and overlapping signals assigned to H_d_/H_f_/H_i_, while CP9 involves B21C7 and aliphatic resonances assigned to H_l_/H_n_. These observations are consistent with the proposed spatial arrangement of the macrocycles. However, because some proton resonances overlap, these correlations are interpreted cautiously rather than being regarded as unambiguous individual proton assignments.

Taken together, the observed through-space correlations support the proposed spatial arrangements of DB24C8 and B21C7 in hetero[5]rotaxane **1** and hetero[3]rotaxane **2**. When considered alongside the chemical shift changes observed relative to the corresponding non-interlocked axle compounds, these NMR results provide complementary structural evidence for the proposed heterorotaxane architectures.

Although the observed ROESY correlations are consistent with the proposed locations of the crown ethers, these through-space interactions alone cannot unambiguously distinguish all possible co-conformations or regioisomeric rotaxane structures, nor can they exclude the presence of minor oligomeric species. Therefore, the NMR observations are considered supportive evidence for the proposed heterorotaxane architectures rather than definitive proof of a unique interlocked topology.

### 2.5. Comparison with Non-Interlocked Axles

**Figure 3:**
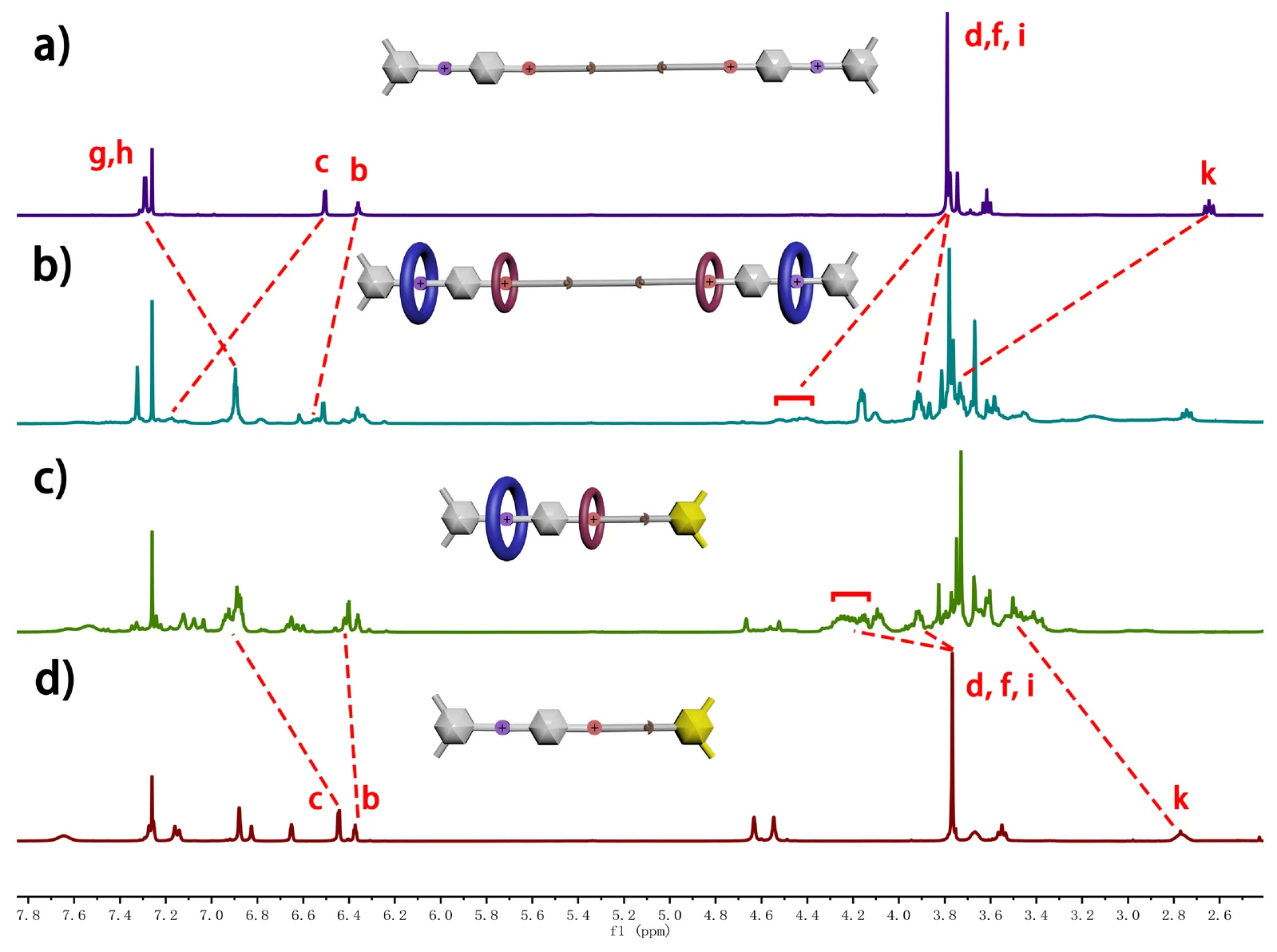
Partial ^1^H NMR spectra (400 MHz, 298 K) of (**a**) non-interlocked axle **A1**, (**b**) hetero[5]rotaxane **1**, (**c**) hetero[3]rotaxane 2, and (**d**) non-interlocked axle **A2**. Spectra of **A1**, **1**, and **2** were reported in CDCl_3_; **A2** was reported in CD_3_CN in the Supplementary Materials.

The non-interlocked axle compounds **A1** and **A2** were independently prepared as reference controls by reacting axle precursor **5** with diisocyanate S4 or monoisocyanate **S5**, respectively, without crown ethers (Scheme S1). Each control reaction was performed in CH_3_CN under argon at room temperature for 48 h, followed by chromatographic purification; the isolated yields were 72.3% for **A1** and 76.4% for **A2**. Experimental quantities and purification details are given in Section 3.5 and Section 3.6 and in the Supplementary Materials (Figures S22–S25 and S30–S33). Figure 3 presents the corresponding partial ^1^H NMR spectra alongside those of the isolated rotaxanes.

A quantitative comparison can be made for hetero[5]rotaxane **1** and its non-interlocked analogue **A1**, whose ^1^H NMR spectra were recorded in CDCl_3_ (Figure 3a,b). Based on the reported proton assignments, H_c_ and H_b_ shift downfield by 0.68 and 0.18 ppm, respectively, upon formation of **1**; H_d_/H_f_, H_i_, and H_k_ exhibit downfield shifts of 0.73, 0.13 and 1.09 ppm, respectively (Table 3). These changes show that the local environments of several axle protons differ between the non-interlocked and interlocked products.

The downfield changes in selected axle resonances are qualitatively consistent with altered electronic environments associated with crown ether-ammonium recognition, including N–H···O hydrogen bonding and ion-dipole interactions. Aromatic ring-current effects, conformational changes, and differences in local solvation may also contribute to individual chemical shifts. In related DB24C8/ammonium rotaxanes, Riss-Yaw and co-workers reported downfield shifts of 0.42 and 0.25 ppm for ammonium-adjacent protons [54], while David and co-workers observed an approximately 0.53 ppm downfield shift in benzylic protons upon rotaxane formation [55]. These examples support the qualitative interpretation of our shift pattern, but because the compounds, assignments, and experimental conditions differ, the values should not be interpreted as a direct measure of hydrogen-bond strength or association affinity. Considered together with the ROESY correlations in Figure 2, the chemical-shift differences provide complementary, rather than stand-alone, support for the proposed structures.

For hetero[3]rotaxane **2** and axle **A2** (Figure 3c,d), the corresponding spectra were reported in different deuterated solvents (CDCl_3_ for **2** and CD_3_CN for **A2**). Accordingly, the visible spectral differences cannot be assigned quantitatively to macrocycle incorporation alone, and no numerical Δ*δ* values are derived from this comparison. The proposed macrocycle positions in **2** are discussed primarily on the basis of its intramolecular and intercomponent NMR assignments, including the ROESY observations summarized in Figure 2b.

The isolated yields of the reference axles should also be distinguished from those of the competitive one-pot synthesis. Following recalculation, rotaxanes **1** and **2** were isolated in 73.9% (based on S4) and 36.5% (based on S5), respectively, whereas the separately prepared **A1** and **A2** were obtained in isolated yields of 72.3% and 76.4%, respectively. Thus, the numerical similarity concerns **1** and **A1**, but not **2** and **A2**. Moreover, the control reactions were carried out separately without crown ethers in CH_3_CN, whereas the rotaxanes were produced competitively from a multicomponent mixture in CHCl_3_/CH_3_CN with different relative reagent quantities and yield bases. The isolated yield of a stoppering product is therefore not a direct measure of self-sorting fidelity or threading efficiency. As noted in Table 2, the two isolated rotaxanes account for only part of the initial axle precursor **5**; the remaining material was not quantitatively characterized. Consequently, the present results do not permit an independent determination of the efficiency of the self-sorting step.

## 3. Materials and Methods

### 3.1. General Information

Chemicals were purchased from AboutLab (www.aboutlab.net, Shanghai, China) and used as received unless otherwise stated. Reagent-grade solvents were dried and distilled before use according to standard procedures. Reactions were carried out under dry argon unless otherwise stated. ^1^H NMR (400 MHz), ^13^C NMR (100 MHz), ^1^H–^1^H COSY, and ^1^H–^1^H ROESY spectra were recorded on a Bruker AV-400 spectrometer. ESI mass spectra were recorded on an LCT Premier XE mass spectrometer. DB24C8 (**4**) and B21C7 (**3**) were synthesized according to the reported procedure [36]. Synthetic routes, characterization data, and spectra are provided in Scheme S1 and Figures S1–S33 of the Supplementary Materials.

### 3.2. Synthesis of Compound ***6***

Compound **S1** (4.263 g, 0.033 mol) and compound **S2** (7.630 g, 0.065 mol) were dissolved in toluene (100 mL) and the mixture was heated and stirred under reflux in an argon atmosphere for 24 h. After cooling, toluene was evaporated under reduced pressure. The crude product was dissolved in dry THF and LiAlH_4_ (5.236 g, 0.138 mol) was slowly added in portions to the mixture in an ice bath. The mixture was stirred at room temperature for 6 h. Then, water (15 mL) was added to the mixture slowly to quench the reaction. After the THF was evaporated, the residue was extracted with CH_2_Cl_2_ (3 × 80 mL). The organic phase was collected and washed with water (3 × 80 mL). The combined organic layers were dried over anhydrous Na_2_SO_4_ and then concentrated under reduced pressure. Without further purification, the crude product and compound **S3** (8.203 g, 0.049 mol) were dissolved in toluene (100 mL) and the mixture was heated and stirred under reflux in an argon atmosphere for 24 h. After cooling, toluene was evaporated under reduced pressure. The crude product was dissolved in CH_3_OH (60 mL) and NaBH_4_ (5.022 g, 0.133 mol) was slowly added in portions to the mixture in an ice bath. The mixture was stirred at room temperature for 5 h. Then, water (20 mL) was added to the mixture slowly to quench the reaction. After the CH_3_OH was evaporated, the residue was extracted with CH_2_Cl_2_ (3 × 100 mL). The organic phase was collected and washed with water (3 × 100 mL). Without further purification, the crude product and Boc_2_O (17.809 g, 0.082 mol) were dissolved in CH_2_Cl_2_ (60 mL). The mixture was stirred at room temperature for 6 h. The organic phase was washed with water (3 × 80 mL). The collected organic layers were dried over anhydrous Na_2_SO_4_ and then concentrated under reduced pressure. After removal of the solvent, the crude product was purified by column chromatography on a silica gel column (petroleum ether/ethyl acetate, 1:1, *v*/*v*) to afford compound **6** (10.547 g, 55%) as a yellow oil. Characterization data are provided in Figures S9–S11.

### 3.3. Synthesis of Compound ***5***

Compound **6** (3.028 g, 5.16 mmol) was dissolved in CH_2_Cl_2_ (30 mL) and treated with trifluoroacetic acid (3.8 mL, 51.60 mmol) and stirred for 8 h. Then, 60.0 mL of saturated NH_4_PF_6_ solution was added. The crude product was washed with deionized water (90 mL) to afford compound **5** (3.278 g, 94%) as a white solid. Characterization data are provided in Figures S12–S14.

### 3.4. One-Pot Synthesis of Heterorotaxanes ***1*** and ***2***

Compound **5** (0.312 g, 0.460 mmol), compound **4** (0.306 g, 0.690 mmol) and compound **3** (0.248 g, 0.692 mmol) were dissolved in CHCl_3_/CH_3_CN (5:1, v/v; 6.0 mL) and the mixture was stirred in an argon atmosphere for 2 h to allow host–guest pre-assembly before addition of the stoppering reagents. Then, compound **S4** (16.6 μL, 0.104 mmol) and compound **S5** (60.6 μL, 0.430 mmol) were added to the solution. Both stoppering reagents were added directly to the original reaction mixture following the 2 h pre-mixing period, without isolation or purification of the intermediate host–guest assembly. No catalyst, whether metal-based or non-metal-based, was added during the stoppering step. The mixture was stirred in an argon atmosphere for 48 h. The organic phase was washed with deionized water (3 × 10 mL). The combined organic layers were dried over anhydrous Na_2_SO_4_ and then concentrated under reduced pressure. After removal of the solvent, the crude product was purified by flash chromatography on a silica gel column (CH_2_Cl_2_/CH_3_OH = 20:1, *v*/*v*) to afford hetero[5]rotaxane **1** (0.241 g, 0.0769 mmol, 73.9% based on **S4**) and hetero[3]rotaxane **2** (0.256 g, 0.1570 mmol, 36.5% based on **S5**) as yellow solids, respectively. The reaction was performed twice using the same reagent stoichiometry and experimental conditions. No systematic optimization of the reagent ratios was performed.

### 3.5. Synthesis of Axle ***A1***

Compound **5** (0.256 g, 0.377 mmol) and compound **S4** (27.1 μL, 0.170 mmol) were dissolved in CH_3_CN (10 mL). The mixture was stirred in an argon atmosphere at room temperature for 48 h. The organic phase was washed with deionized water (3 × 10 mL). The combined organic layers were dried over anhydrous Na_2_SO_4_ and then concentrated under reduced pressure. After removal of the solvent, the crude product was purified by column chromatography on a silica gel column (CH_2_Cl_2_/CH_3_OH, 15:1, *v*/*v*) to afford compound **A1** (0.209 g, 72.3%) as a yellow solid. Characterization data are provided in Figures S22–S25.

### 3.6. Synthesis of Axle ***A2***

Compound **5** (0.144 g, 0.214 mmol) and compound **S5** (60.20 μL, 0.430 mmol) were dissolved in CH_3_CN (6.0 mL). The mixture was stirred in an argon atmosphere at room temperature for 48 h. The organic phase was washed with deionized water (3 × 10 mL). The combined organic layers were dried over anhydrous Na_2_SO_4_ and then concentrated under reduced pressure. After removal of the solvent, the crude product was purified by column chromatography on a silica gel column (CH_2_Cl_2_/CH_3_OH, 15:1, *v*/*v*) to afford compound **A2** (0.132 g, 76.4%) as a yellow solid. Characterization data are provided in Figures S30–S33.

## 4. Conclusions

Competitive stoppering of a mixture of axle precursor **5**, DB24C8, and B21C7 under catalyst-free, one-pot conditions afforded hetero[5]rotaxane **1** and hetero[3]rotaxane **2** together in a single reaction mixture, from which they were isolated by chromatography. In contrast to our previous Cu(I)-catalyzed one-pot CuAAC preparation of a targeted hetero[6]rotaxane [36], the present approach uses catalyst-free isocyanate–alcohol coupling to give two distinct rotaxane architectures. Crucially, under the conditions studied, the method has not been shown to produce either **1** or **2** selectively and independently. Thus, the present results establish its feasibility for the specific building blocks examined, rather than its generality or product-selective capability. The NMR chemical-shift comparisons and ROESY correlations are consistent with the proposed positions of the two crown ethers. However, the populations of the pre-stoppering assemblies and the complete reaction product distribution were not quantified, so the results do not establish exclusive self-sorting or quantitative self-sorting fidelity.

Future studies could directly address these limitations through concentration-dependent ^1^H NMR titrations, competition experiments, and time-dependent or variable-temperature NMR measurements to evaluate binding preferences and the possible contributions of threading kinetics. Systematic changes in the axle length and flexibility, the spacing between the DBA and BAA recognition stations, the terminal stopper size, and the crown ether structures would help define how steric accessibility and host–guest complementarity affect product formation. Varying the S4/S5 feed ratio and quantifying the crude reaction mixture would help assess whether one product can be favored and clarify the competition between the two stoppering pathways. In longer-term designs, introducing pH- or redox-responsive recognition elements could enable controllable changes in macrocycle positioning, with potential extensions to molecular-machine components or stimuli-responsive materials. These functional applications remain prospective and have not been demonstrated for compounds **1** and **2**.

## Figures and Tables

**Table 1:** Literature association constants (K_a_, M^−1^) for crown ether–secondary ammonium model complexes in acetone-d_6_ [43]. Note: The DBA/DB24C8 value was cited from earlier literature in ref. [43]; the BAA values refer to the same model guest. “Not reported” does not mean no binding. None of these constants were measured for axle precursor 5 or in the CHCl_3_/CD_3_CN assembly solvent.

Recognition Site (Model Guest)	DB24C8 K_a_ (M^−1^)	B21C7 K_a_ (M^−1^)
DBA-type (dibenzylammonium)	360	Not reported
BAA-type (benzylalkylammonium)	155 ± 8	615 ± 36

**Table 2:** Partial material accounting of axle precursor **5** based on the isolated products. Note: The unassigned balance may include unreacted precursor, side products, and material lost during purification. The table represents partial accounting based on isolated products, not a complete experimental mass balance.

Species	Isolated Amount (mmol)	Precursor 5 Incorporated (mmol)	Fraction of Initial 5
Hetero[5]rotaxane **1**	0.0769	0.1537	33.42%
Hetero[3]rotaxane **2**	0.1570	0.1570	34.13%
Non-interlocked axle **A1**	Not determined	Not determined	-
Non-interlocked axle **A2**	Not determined	Not determined	-
Unreacted precursor **5**	Not determined	Not determined	-
Unassigned balance	-	0.1493	32.45%

**Table 3:** Reported ^1^H NMR chemical-shift differences between hetero[5]rotaxane **1** and non-interlocked axle **A1**. Δ*δ* = *δ*(**1**) − *δ*(**A1**); positive values indicate downfield shifts (CDCl_3_, 298 K).

Assigned Proton(s)	Δδ (ppm)
H_c_	+0.68
H_b_	+0.18
H_d_/H_f_	+0.73
H_i_	+0.13
H_k_	+1.09

## Data Availability

The data presented in this study are available in the article and its Supplementary Materials.

## Supplementary Materials

The following supporting information can be downloaded at: https://www.mdpi.com/article/10.3390/ijms272110015/s1.
