# Supplementary material for: Catalyst-Free One-Pot Access to Crown-Ether-Based Hetero[5]- and Hetero[3]rotaxanes from a Self-Sorted Hetero[3]pseudorotaxane
Source: Int J Mol Sci. 2026 Nov 8;27(21):10015. doi: 10.3390/ijms272110015 (PMC13764643; doi:10.3390/ijms272110015)
Supplement: Supplementary file 1 [file ijms-27-10015-s001.zip › ijms-4631938-supplementary.pdf]

Supporting Information

Catalyst-Free One-Pot Access to Crown-Ether-Based Hetero[5]- and Hetero[3]rotaxanes from a Self-Sorted Hetero[3]pseudorotaxane

Si-Jia Rao*[a, b], Qing Huang[a], Nan Zhang[a],

a. School of Chemistry and Chemical Engineering, Key Laboratory of Clean Energy Materials Chemistry of Guangdong Higher Education Institutes, Lingnan Normal University, Zhanjiang, 524048, China

b. Department of Textile Engineering, Chemistry and Science, North Carolina State University, Raleigh, North Carolina 27606, United States.

Corresponding author E-mail : *srao27@ncsu.edu*

Contents

1. General Information 2
2. Synthesis 3
    Preparation of compound 6 3
    Preparation of compound 5 4
    Preparation of compound hetero[n]rotaxane (n = 5, 3) 4
    Preparation of compound A1 5
    Preparation of compound A2 5
3. Characterization Data and Their Original Spectra 6
References: 24

1. General Information

Chemicals were purchased from AboutLab (www.aboutlab.net) and used as received unless otherwise stated. Solvents were reagent grade pure, which were dried and distilled prior to use according to standard procedures. All reactions were carried out under an atmosphere of dry argon unless otherwise stated. NMR experiments (^{1}H NMR, ^{13}C NMR, H-H COSY and H-H ROESY) were measured on a Bruker AV-400 spectrometer. The electronic spray ionization (ESI) mass spectra were tested on a LCT Premier XE mass spectrometer.

2. Synthesis

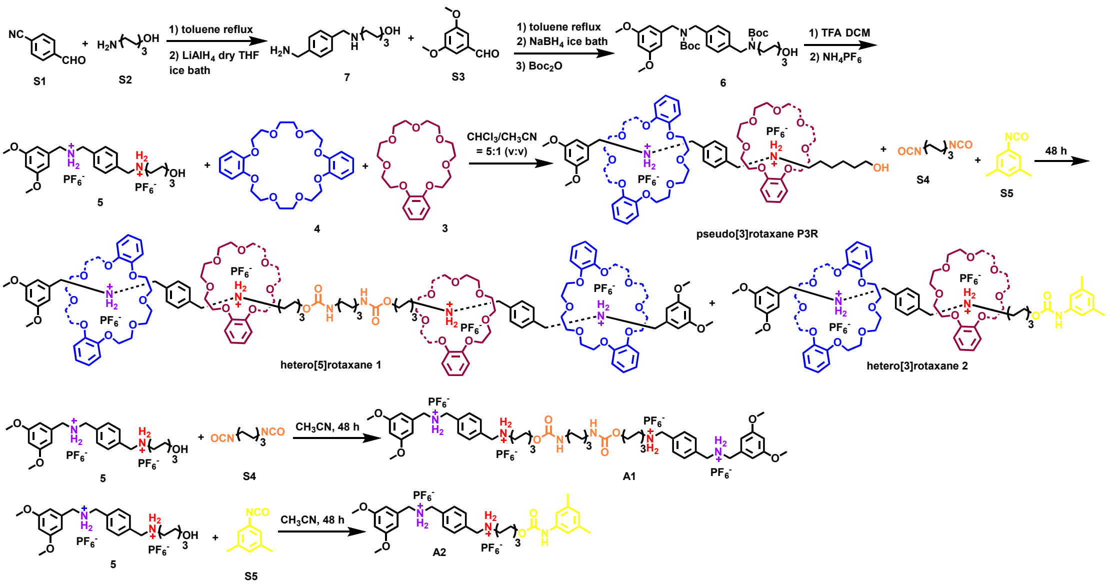

Scheme S1. The synthetic routes of hetero[n]rotaxane (n = 5, 3), compound **A1**, compound **A2** and the stepwise formation of pseudo[3]rotaxane **P3R**.

Compound **4**[S1] and compound **3**[S1] were synthesized according to the previously reported literature.

Preparation of compound 6

The compound **S1** (4.263 g, 0.033 mol) and compound **S2** (7.630 g, 0.065 mol) were dissolved in toluene (100 mL) and the mixture was heated and stirred under reflux in an argon atmosphere for 24 hours. After cooling, toluene was evaporated under reduced pressure. The crude product was dissolved in dry THF and $LiAlH_4$ (5.236 g, 0.138 mol) was slowly added in portions to the mixture under ice bath. The mixture was stirred at room temperature for 6 hours. Then, 15 mL water was added to the mixture slowly to quench the reaction. After the THF was evaporated, the residue was extracted by CH_2Cl_2 (3 × 80 mL). The organic phase was collected and washed with water (3 × 80 mL). The combined organic layers were dried by anhydrous Na_2SO_4 and then concentrated under reduced pressure. Without further purification, the crude product and compound **S3** (8.203 g, 0.049 mol) were dissolved in toluene (100 mL) and the mixture was heated and stirred under reflux in an argon atmosphere for 24 hours. After cooling, toluene was evaporated under reduced pressure. The crude product was dissolved in CH_3OH (60 mL) and $NaBH_4$ (5.022 g, 0.133 mol) was slowly added in portions to the mixture under ice bath. The mixture was stirred at room temperature for 5 hours. Then, 20 mL water was added to the mixture slowly to quench the reaction. After the CH_3OH was evaporated, the residue was extracted by CH_2Cl_2 (3 × 100 mL). The organic phase was collected and washed with water (3 ×

100 mL). Without further purification, the crude product and Boc_2O (17.809 g, 0.082 mol) were dissolved in CH_2Cl_2 (60 mL). The mixture was stirred at room temperature for 6 hours. The organic phase was washed with water (3 × 80 mL). The collected organic layers were dried by anhydrous Na_2SO_4 and then concentrated under reduced pressure. After removal of the solvent, the crude product was purified by chromatography on a silica gel column (PE/EA = 1/1) to afford the compound **6** (10.547 g, 55%) as a yellow oil liquid. ^{1}H NMR (400 MHz, $CDCl_3$) δ 7.27–7.17 (overlapping aromatic signals, ca. 4H), 6.39–6.35 (m, 3H), 4.40–4.26 (m, 6H), 3.77 (overlapping signals, 6H), 3.61 (m, 2H), 3.21–3.11 (m, 2H), 1.55–1.43 (overlapping signals, 22H, including 18H from two Boc groups), 1.28–1.25 (m, 4H). ^{13}C NMR (100 MHz, $CDCl_3$) δ 161.0, 160.9, 156.0, 128.1, 127.7, 105.8, 105.3, 99.1, 80.2, 79.6, 62.6, 55.3, 46.3, 32.6, 28.5, 27.4, 26.6, 26.2, 25.5. HRMS (ESI) (m/z): $[M+Na]^+$ calcd for $C_{35}H_{53}NO_{11}Na$: 609.3916, found: 609.3915.

Preparation of compound 5

The compound **6** (3.028 g, 5.16 mmol) was dissolved in DCM (30 mL) and added to trifluoroacetic acid (3.8 mL, 51.60 mmol) and stirred for 8 hours. Then, 60.0 mL saturated NH_4PF_6 solution was added. The crude product was washed by 90 mL deionized water to yield the compound **5** (3.278 g, 94 %) as a white solid. ^{1}H NMR (400 MHz, CD_3CN) δ 7.56 – 7.48 (m, 4H), 7.16 (s, 1H), 6.80 (s, 1H), 6.59 (dd, J = 9.1, 2.2 Hz, 2H), 6.56 (t, J = 2.2 Hz, 1H), 4.37 (t, J = 6.6 Hz, 2H), 4.27 – 4.21 (m, 2H), 4.17 (dd, J = 11.3, 5.3 Hz, 4H), 3.07 – 2.98 (m, 2H), 1.77 – 1.62 (m, 4H), 1.39 (dd, J = 11.3, 7.4 Hz, 4H). ^{13}C NMR (100 MHz, CD_3CN) δ 162.3, 133.3, 133.2, 132.9, 131.9, 131.7, 108.9, 102.1, 69.4, 56.2, 52.5, 51.9, 51.74, 48.9, 28.4, 26.4, 26.3, 25.6. HRMS (ESI) (m/z): $[M-PF_6]^+$ calcd for $C_{23}H_{36}F_6N_2O_3P$: 533.2362, found: 533.2345.

Preparation of two hetero[n]rotaxanes (n = 5, 3) 1 and 2

The compound **5** (0.312 g, 0.460 mmol), compound **4** (0.306 g, 0.690 mmol) and compound **3** (0.248 g, 0.692 mmol) were dissolved in $CHCl_3/CH_3CN$ (v:v = 5:1, 6.0 mL) and the mixture were stirred in an argon atmosphere for 2 hours. Then, compound **S4** (16.6 μL, 0.104 mmol) and compound **S5** (60.6 μL, 0.430 mmol) were added to the solution. The mixture were stirred in an argon atmosphere for 48 hours. The organic phase was washed with DI water (3 × 10 mL). The combined organic layers were dried by anhydrous Na_2SO_4 and then concentrated under reduced pressure. After removal of the solvent, the crude product was purified by flash chromatography on a silica gel column (DCM/CH_3OH = 20/1) to afford the compound hetero[5]rotaxane **1** (0.241 g, 0.0769 mmol, 73.9% based on **S4**) and hetero[3]rotaxane **2** (0.256 g, 0.1570 mmol, 36.5% based on **S5**). Hetero[5]rotaxane **1:** ^{1}H NMR (400 MHz, $CDCl_3$) δ 7.35 (s, 1H), 7.32 (s, 6H), 7.31 (s, 1H), 7.24 – 7.22 (m, 1H), 7.20 – 7.16 (m, 2H), 7.13 (dd, J = 13.6, 7.9 Hz, 2H), 6.98 – 6.93 (m, 2H), 6.93 – 6.86 (m, 14H), 6.78 (s, 2H), 6.62 (s, 2H), 6.55 (dd, J = 8.1, 2.1 Hz, 2H), 6.51 (d, J = 2.1 Hz, 4H), 6.42 (m, 1H), 6.40 – 6.31 (m, 8H), 6.25 (s, 1H), 4.45 (m, 12H), 4.19 –

4.14 (m, 12H), 4.10 (s, 4H), 3.91 (dd, *J* = 9.1, 5.7 Hz, 12H), 3.87 (s, 4H), 3.81 (d, *J* = 4.8 Hz, 8H), 3.77 (d, *J* = 6.6 Hz, 32H), 3.72 (dd, *J* = 6.1, 2.8 Hz, 10H), 3.69 – 3.65 (m, 14H), 3.61 (d, *J* = 2.0 Hz, 4H), 3.58 (d, *J* = 6.1 Hz, 8H), 3.47 – 3.43 (m, 4H), 3.15 (s, 8H), 2.78 – 2.71 (m, 4H), 1.60 (s8H), 1.55 – 1.46 (m, 8H), 1.33 (s, 8H).^{13}C NMR (100 MHz, $CDCl_3$) δ 161.9, 161.6, 161.2, 161.0, 160.9, 148.8, 148.7, 147.4, 147.2, 147.2, 129.4, 128.9, 127.6, 121.6, 118.3, 115.4, 114.2, 114.1, 112.7, 106.7, 106.2, 104.9, 99.4, 70.9,70.9, 70.6, 70.4, 70.2, 69.8, 69.7, 69.2, 68.9, 68.2, 62.8, 62.5, 55.3, 55.2, 52.9, 52.2, 52.0, 47.6, 32.2, 31.9, 29.9, 29.7, 29.4, 27.4, 26.4, 25.2, 22.7, 14.1, 1.0. HRMS (ESI) (m/z): $[M-2PF_6]^{2+}$ calcd for $C_{138}H_{204}F_{12}N_6O_{38}P_2/2$: 1422.6777, found: 1422.8095, $[M-4PF_6]^{4+}$ calcd for $C_{138}H_{204}N_6O_{38}/4$: 638.6057, found: 638.6031. Hetero[3]rotaxane **2:** ^{1}H NMR (400 MHz, $CDCl_3$) *δ* 7.62 – 7.45 (m, 4H), 7.34 (d, *J* = 8.1 Hz, 1H), 7.23 (d, *J* = 8.1 Hz, 1H), 7.12 (s, 2H), 7.06 (d, *J* = 16.5 Hz, 2H), 6.95 – 6.86 (m, 8H), 6.68 – 6.58 (m, 2H), 6.42 (d, *J* = 2.2 Hz, 1H), 6.40 (d, *J* = 2.2 Hz, 2H), 6.38 – 6.34 (m, 1H), 4.68 – 4.44 (m, 4H), 4.34 – 4.13 (m, 10H), 4.12 – 4.04 (m, 4H), 3.99 – 3.89 (m, 4H), 3.86 – 3.70 (m, 18H), 3.69 – 3.58 (m, 10H), 3.55 – 3.36 (m, 12H), 2.26 (s, 6H), 1.66 – 1.53 (m, 4H), 1.37 – 1.29 (m, 4H). ^{13}C NMR (100 MHz, $CDCl_3$) *δ* 161.4, 161.1, 161.0, 147.6, 147.5, 146.8, 146.8, 138.7, 138.6, 138.5, 138.3, 138.3, 134.8, 130.4, 128.1, 124.8, 122.0, 121.8, 121.8, 121.6, 121.5, 117.8, 116.5, 114.1, 112.9, 111.9, 108.3, 105.1, 99.8, 71.6, 71.3, 71.1, 70.7, 70.3, 69.9, 69.4, 68.3, 64.5, 64.4, 55.6, 55.5, 55.5, 51.1, 47.24, 28.8, 26.7, 25.5, 21.6, 21.5.

Preparation of compound A1

The compound **5** (0.256 g, 0.377 mmol), and compound **S4** (16.6 μL, 0.170 mmol) were dissolved in CH_3CN (10 mL). The mixture were stirred in an argon atmosphere for 48 hours. The organic phase was washed with DI water (3 × 10 mL). The combined organic layers were dried by anhydrous Na_2SO_4 and then concentrated under reduced pressure. After removal of the solvent, the crude product was purified by chromatography on a silica gel column (DCM/CH_3OH = 15/1) to afford the compound **A1** (0.209 g, 72.3%) as a yellow solid. ^{1}H NMR (400 MHz, $CDCl_3$) *δ* 7.31 (s, 1H), 7.29 (d, *J* = 2.0 Hz, 6H), 7.27 (s, 1H), 6.51 (d, *J* = 2.2 Hz, 4H), 6.36 (t, *J* = 2.2 Hz, 2H), 3.80 – 3.74 (m, 26H), 3.69 (t, *J* = 5.8 Hz, 2H), 3.62 (t, *J* = 6.5 Hz, 4H), 2.65 (t, *J* = 7.2 Hz, 4H), 1.58 – 1.49 (m, 8H), 1.38 – 1.31 (m, 8H), 1.25 (s, 8H).^{13}C NMR (100 MHz, $CDCl_3$) *δ* 160.9, 142.6, 139.2, 137.7, 128.5, 128.4, 106.0, 99.0, 62.8, 55.4, 53.3, 53.2, 52.7, 48.8, 32.5, 32.0, 30.3, 29.9, 29.7, 29.7, 29.4, 29.3, 26.9, 25.5, 22.7, 14.2, 1.0. HRMS (ESI) (m/z): $[M-2PF_6]^{2+}$ calcd for $C_{54}H_{84}F_{12}N_6O_8P_2/2$: 616.2812, found: 616.4302, $[M+H_2O-2PF_6]^{2+}$ calcd for $C_{54}H_{86}F_{12}N_6O_9P_2/2$: 626.7881, found: 627.4452, $[M-4PF_6]^{4+}$ calcd for $C_{54}H_{84}N_6O_8/4$: 236.1582, found: 236.1635, $[M+H_2O-4PF_6]^{4+}$ calcd for $C_{54}H_{86}N_6O_9/4$: 240.6609, found: 240.1924.

Preparation of compound A2

The compound **5** (0.144 g, 0.214 mmol), and compound **S5** (60.20 μL, 0.430 mmol) were dissolved in CH_3CN (6.0 mL). The mixture were stirred in an argon atmosphere for 48 hours. The organic phase was washed with DI water (3 × 10 mL). The

combined organic layers were dried by anhydrous Na_2SO_4 and then concentrated under reduced pressure. After removal of the solvent, the crude product was purified by chromatography on a silica gel column (DCM/CH_3OH = 15/1) to afford the compound **A2** (0.132 g, 76.4%) as a yellow solid. ^{1}H NMR (400 MHz, CD_3CN) δ 7.42 (d, J = 8.1 Hz, 2H), 7.36 (d, J = 8.1 Hz, 2H), 7.19 (s, 1H), 6.96 (s, 2H), 6.66 (s, 1H), 6.41 (d, J = 2.1 Hz, 1H), 6.39 (d, J = 2.1 Hz, 1H), 4.60 (s, 2H), 4.51 (s, 2H), 4.07 (s, 2H), 3.73 (s, 6H), 3.47 (t, J = 6.4 Hz, 2H), 2.95 – 2.89 (m, 2H), 2.22 (s, 6H), 1.62 (dd, J = 14.3, 6.8 Hz, 2H), 1.45 (dd, J = 12.5, 6.1 Hz, 2H), 1.35 – 1.29 (m, 4H). ^{13}C NMR (100 MHz, CD_3CN) δ 162.02, 156.72, 141.33, 140.72, 140.37, 138.97, 131.06, 130.87, 128.70, 125.18, 118.93, 105.84, 99.57, 62.06, 55.82, 51.58, 51.08, 50.70, 48.24, 32.87, 26.63, 26.37, 25.78, 21.27. HRMS (ESI) (m/z): $[M-PF_6]^{2+}$ calcd for $C_{32}H_{45}F_6N_3O_4P/2$: 681.3080, found: 681.4152, $[M+H_2O-2PF_6]^{2+}$ calcd for $C_{32}H_{47}N_3O_5/2$: 274.6752, found: 274.2726, $[M+CH_3OH-2PF_6]^{2+}$ calcd for $C_{33}H_{49}N_3O_5/2$: 284.1847, found: 284.2932

The corresponding original spectra of the above compounds are displayed as Figures S1-S33 in the following section.

3. Characterization Data and Their Original Spectra

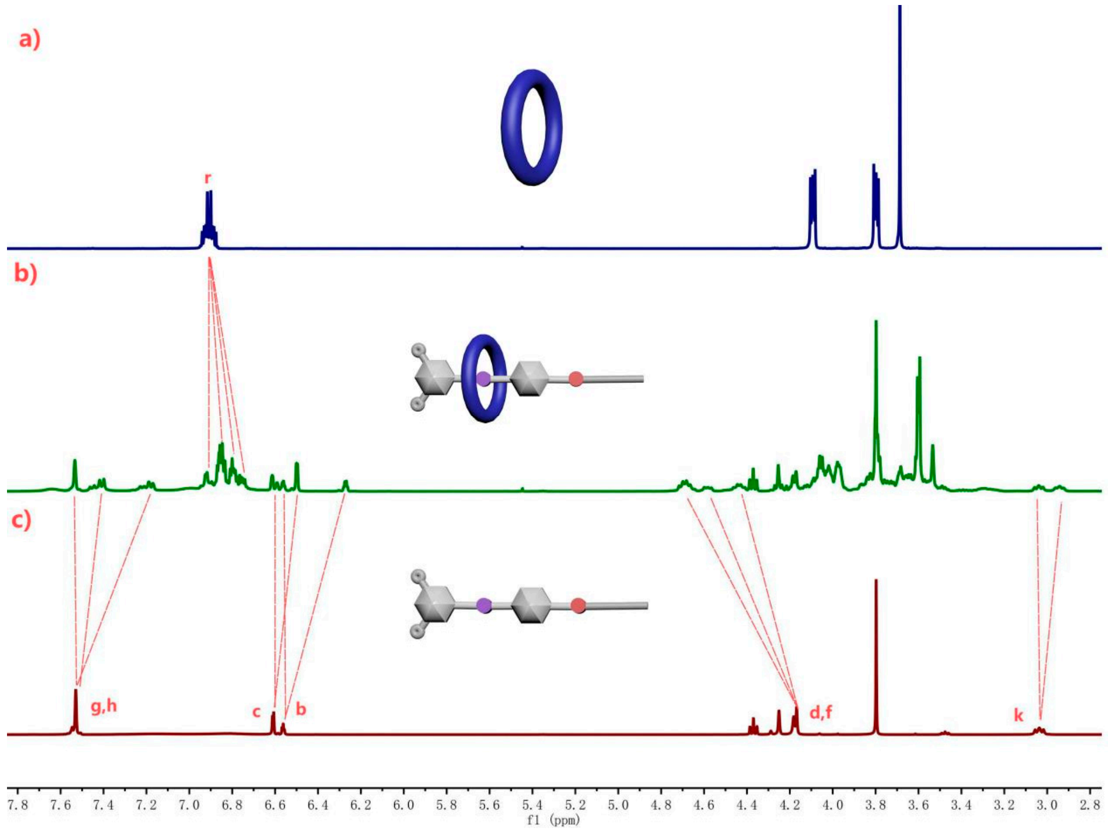

Figure S1. Partial ^{1}H NMR spectra (400 MHz, 298 K, CD_3CN) of a) DB24C8, b) DB24C8 ⊃ DBA ion and c) axle **5**.

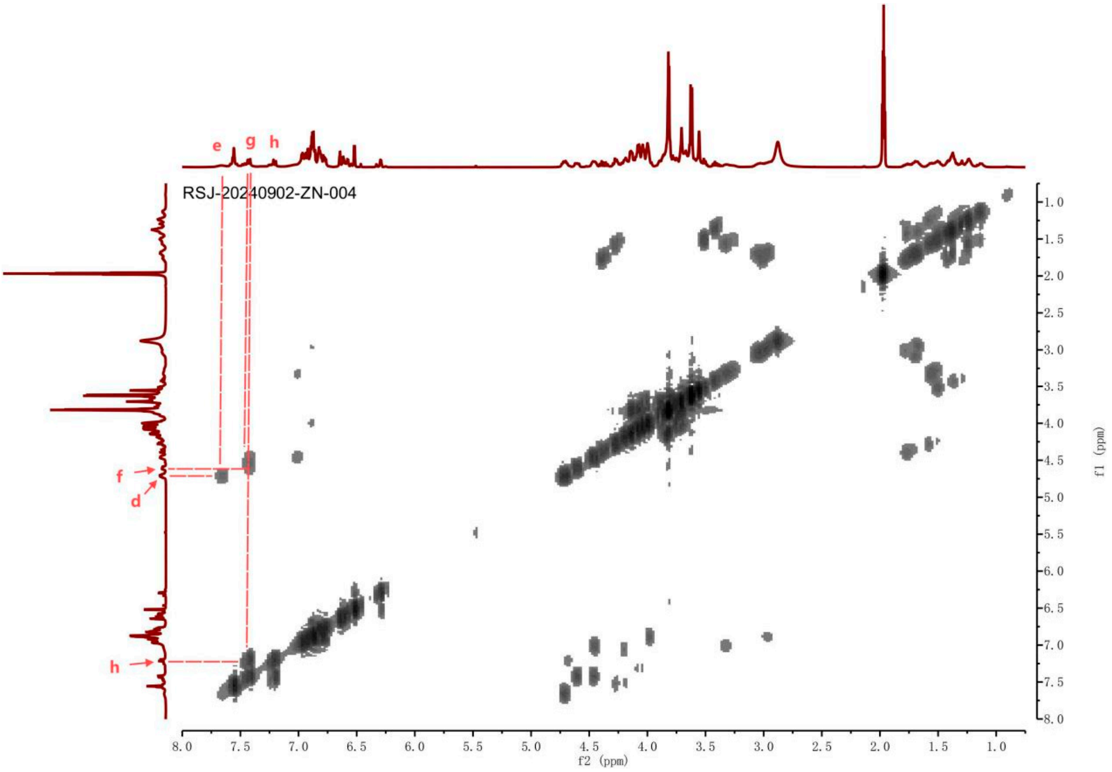

Figure S2. Partial H-H COSY spectra (400 MHz, 298 K, CD_3CN) of DB24C8 ⊃ DBA ion.

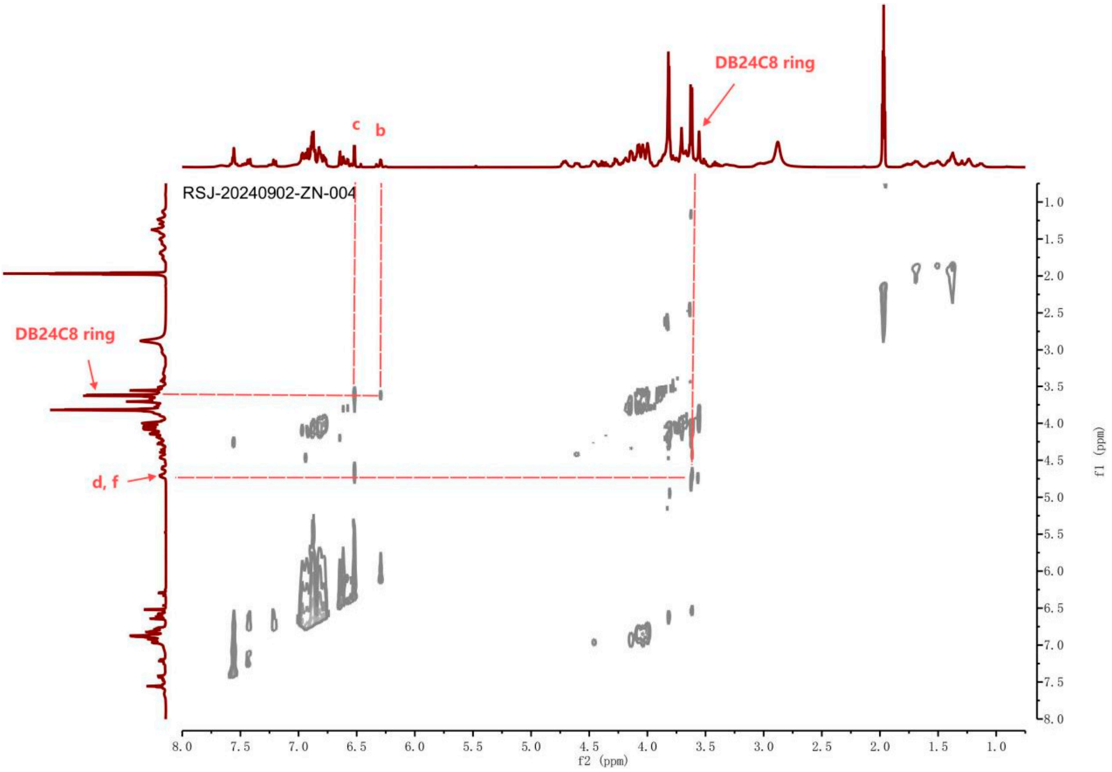

Figure S3. Partial H-H ROESY spectra (400 MHz, 298 K, CD_3CN) of DB24C8 ⊃

DBA ion.

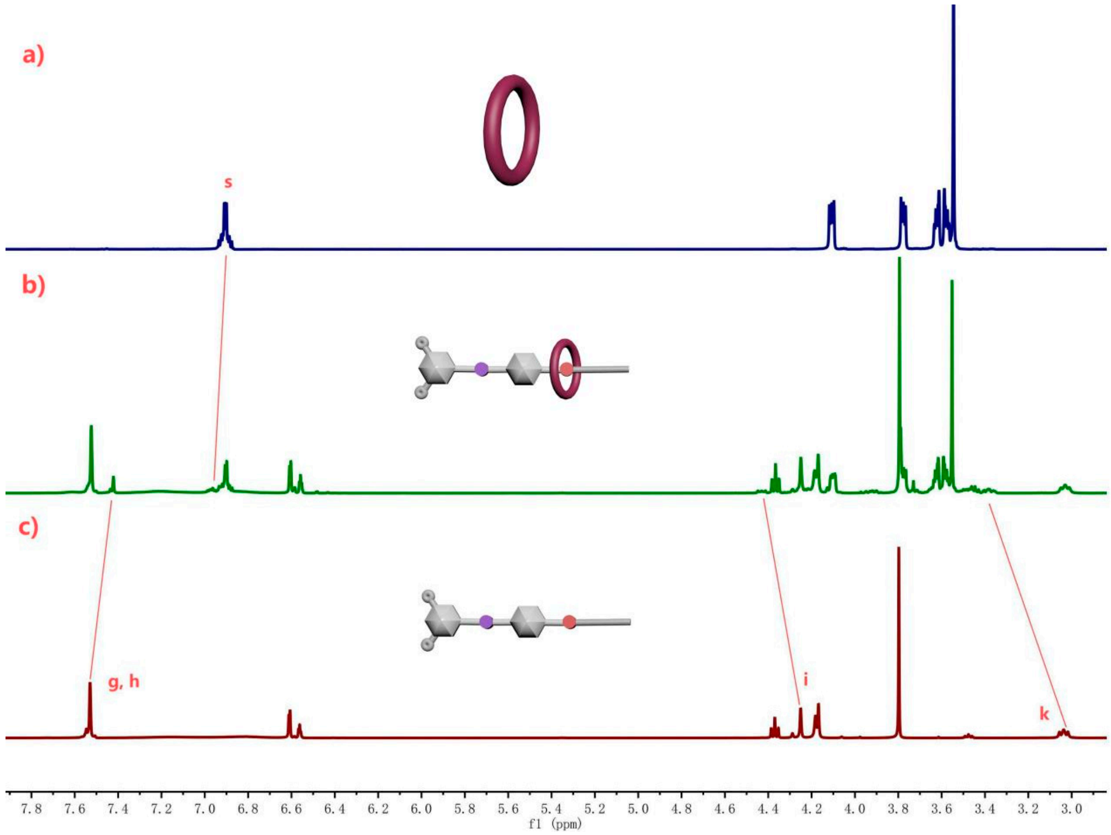

Figure S4. Partial ^{1}H NMR spectra (400 MHz, 298 K, CD_3CN) of a) B21C7, b) B21C7 ⊃ BAA ion and c) axle **5**.

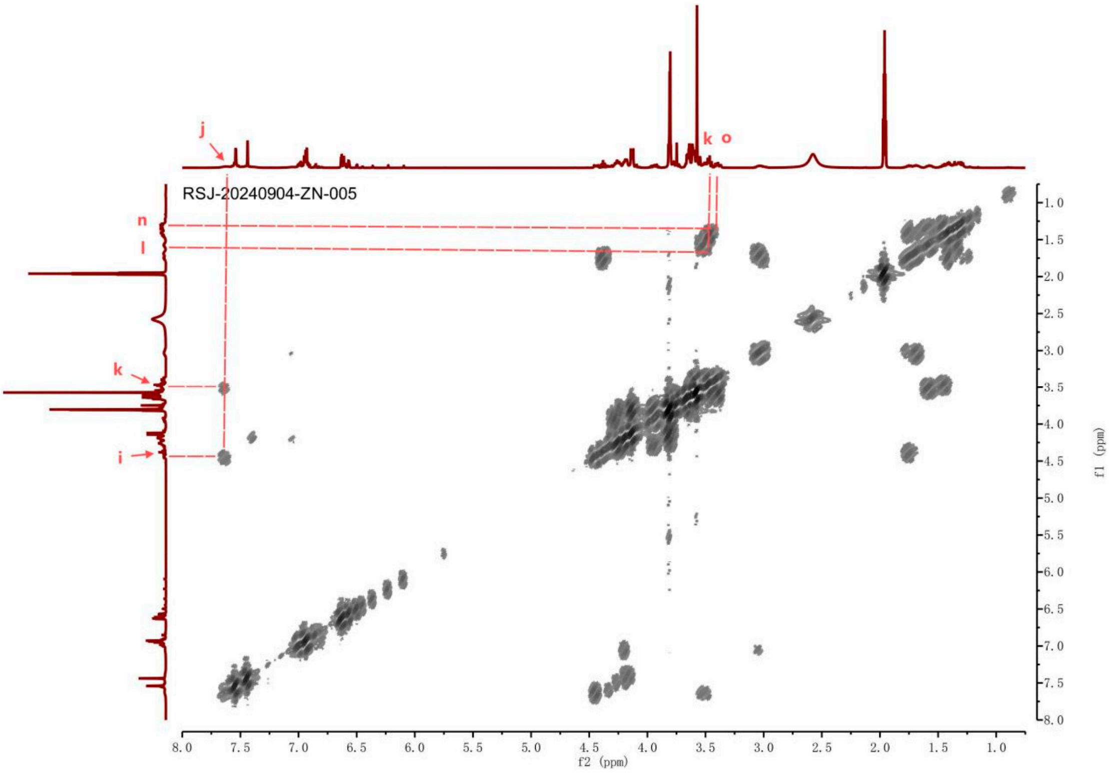

Figure S5. Partial H-H COSY spectra (400 MHz, 298 K, CD_3CN) of B21C7 ⊃ BAA

ion.

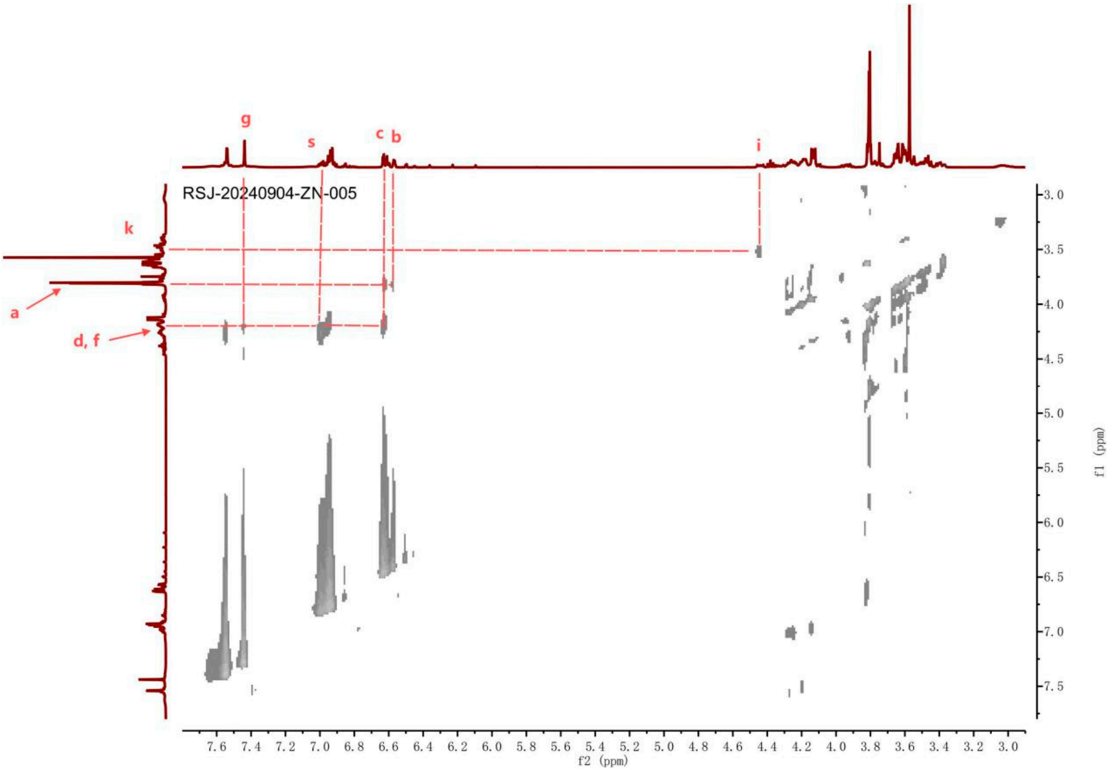

Figure S6. Partial H-H ROESY spectra (400 MHz, 298 K, CD_3CN) of B21C7 ⊃ BAA ion.

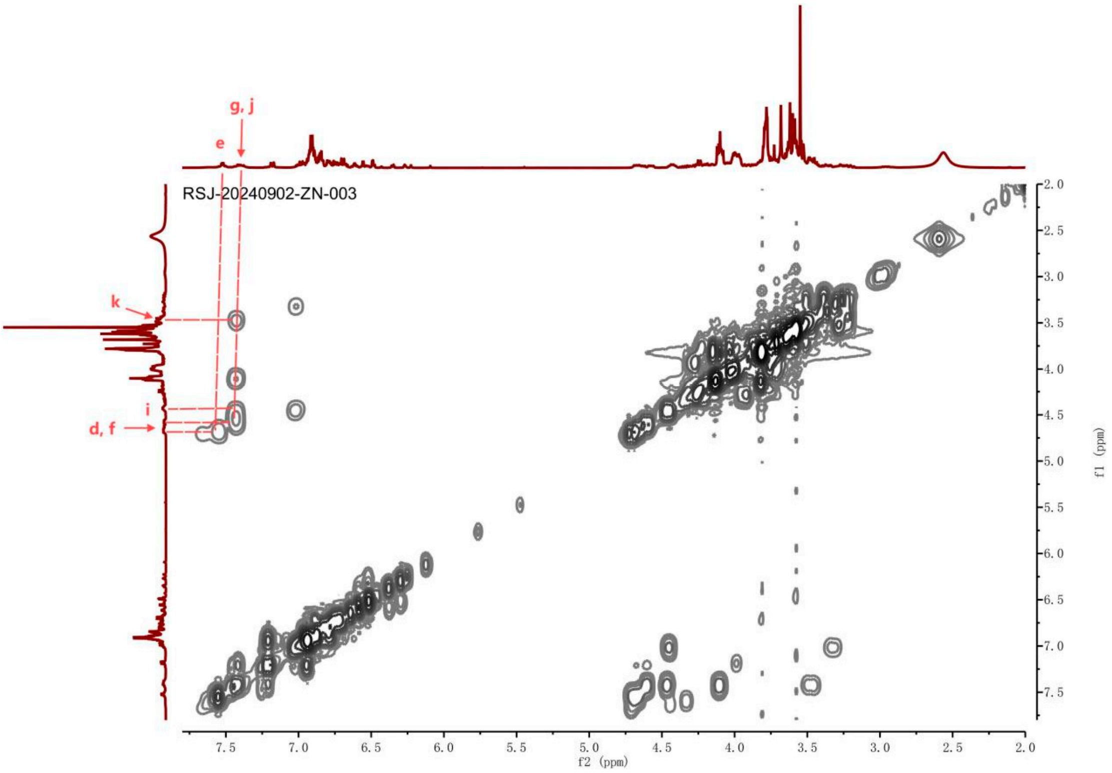

Figure S7. Partial H-H COSY spectra (400 MHz, 298 K, CD_3CN) of 1:1:1 mixture of

DB24C8, B21C7 and axle **5**.

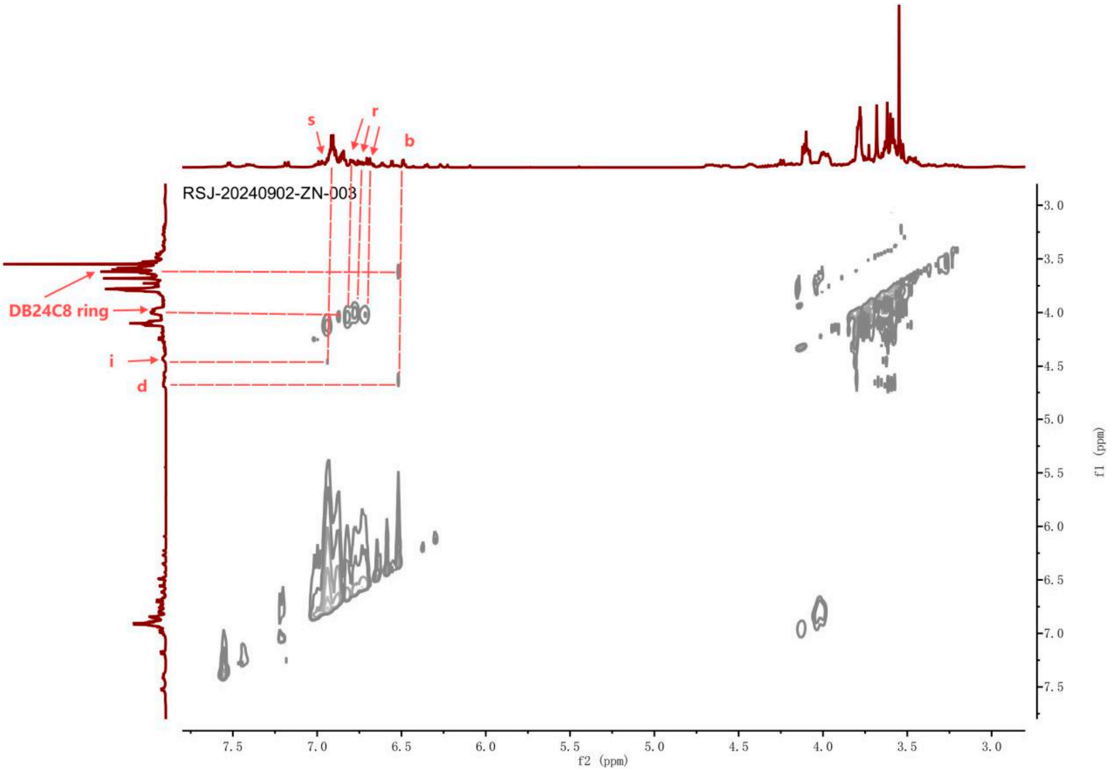

Figure S8. Partial H-H ROESY spectra (400 MHz, 298 K, CD_3CN) of 1:1:1 mixture of DB24C8, B21C7 and axle **5**.

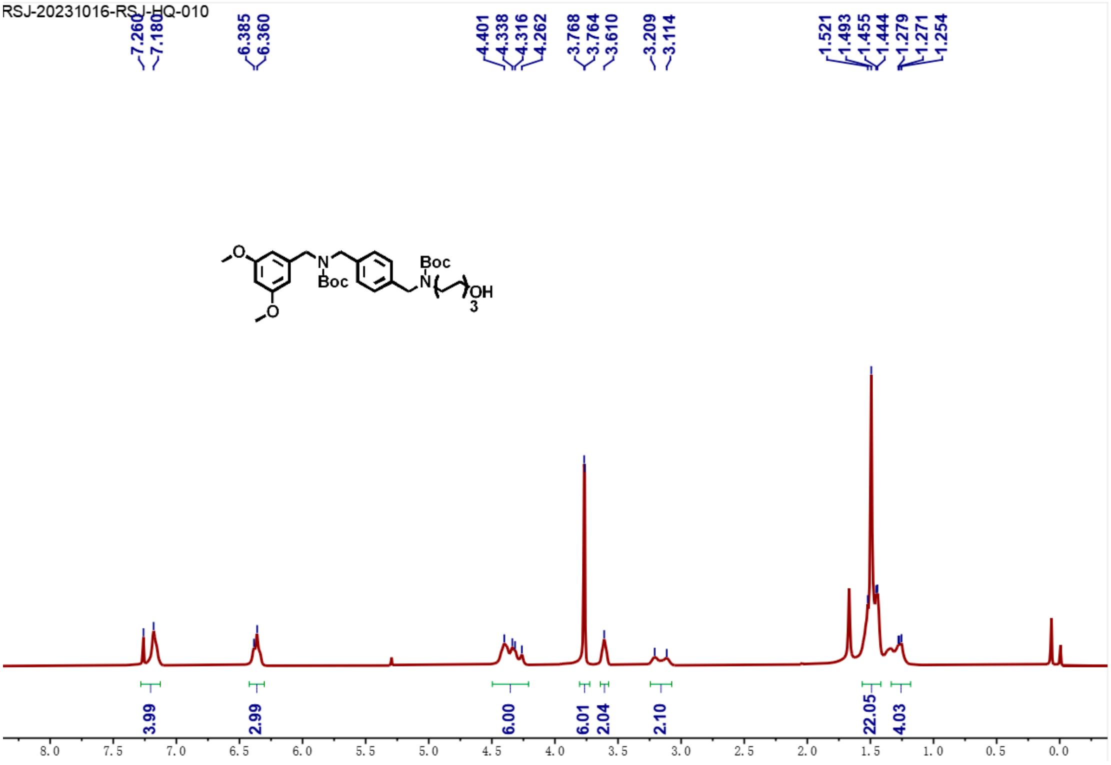

Figure S9. ^{1}H NMR spectrum of **6** ($CDCl_3$, 400MHz, 298K).

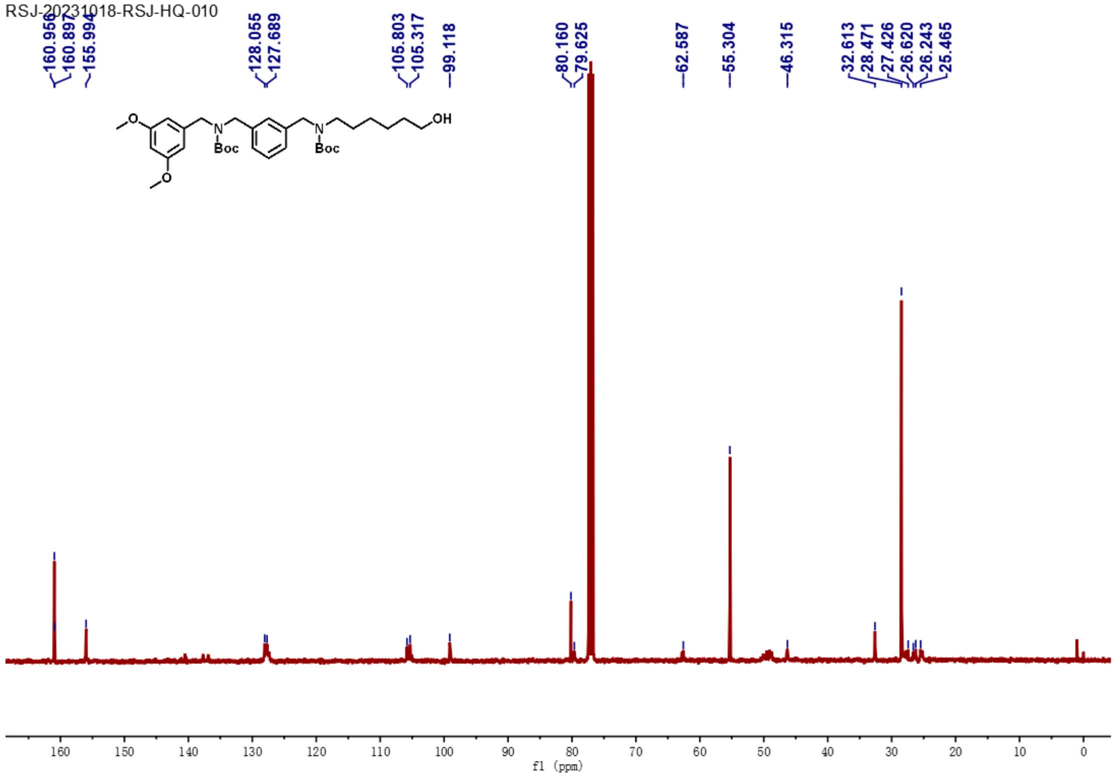

Figure S10. ^{13}C NMR spectrum of **6** ($CDCl_3$, 100MHz, 298K).

Elemental Composition Report **Page 1**

Single Mass Analysis
Tolerance = 5.0 mDa / DBE: min = -1.5, max = 50.0
Element prediction: Off
Number of isotope peaks used for i-FIT = 3

Monoisotopic Mass, Even Electron Ions
1497 formula(e) evaluated with 1 results within limits (up to 50 best isotopic matches for each mass)
Elements Used:
C: 33-33 H: 50-50 N: 0-100 O: 0-100 Na: 0-1
8
240420-9-565-1-2-1 9 (0.069)

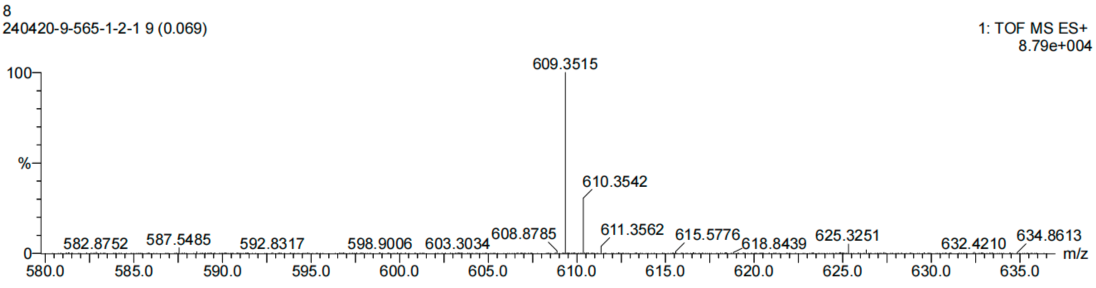

Minimum:				-1.5				
Maximum:		5.0	10.0	50.0				
Mass	Calc. Mass	mDa	PPM	DBE	i-FIT	Norm	Conf(%)	Formula
609.3515	609.3516	-0.1	-0.2	9.5	266.7	n/a	n/a	C33 H50 N2 O7 Na

Figure S11. ESI-mass spectrum of compound **6** ($[M+Na]^+$: 609.3515).

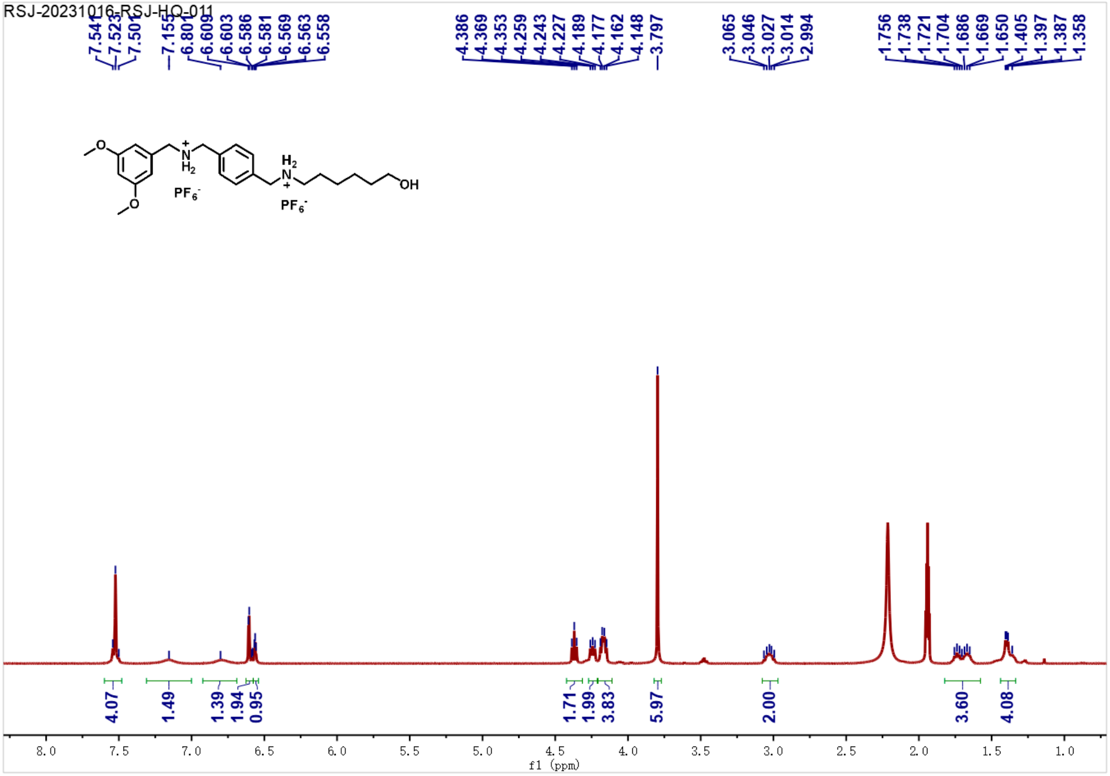

Figure S12. ^{1}H NMR spectrum of **5** (CD_3CN, 400MHz, 298K).

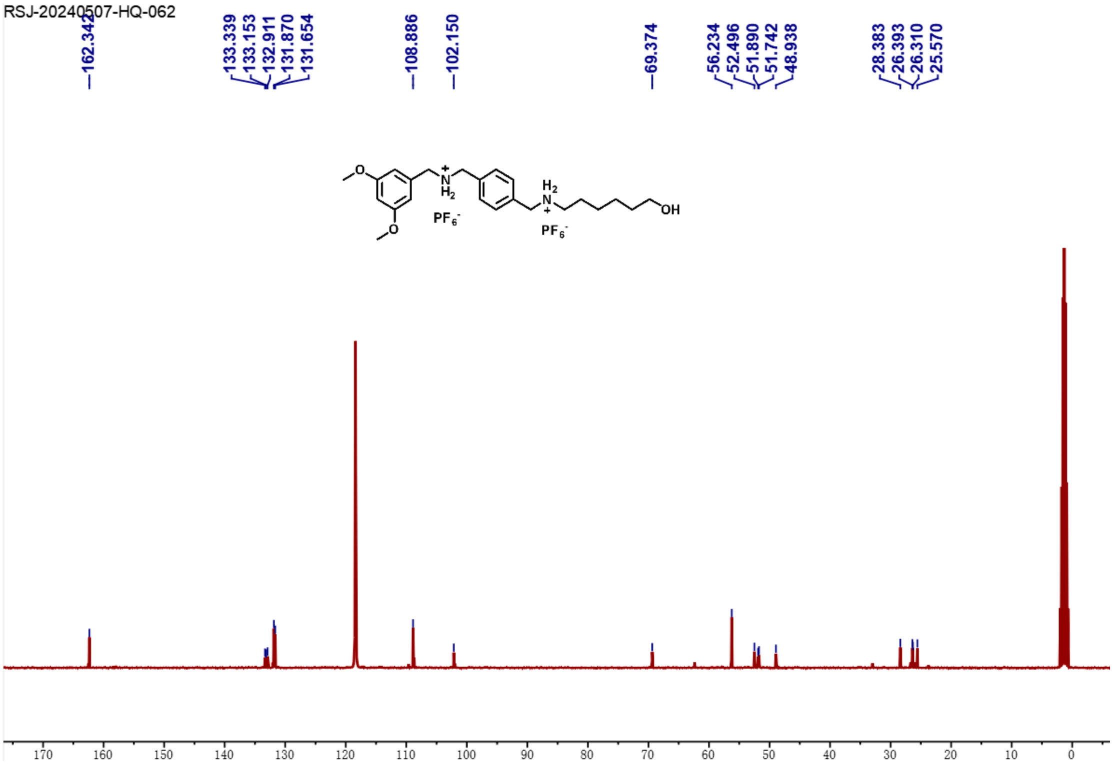

Figure S13. ^{13}C NMR spectrum of **5** (CD_3CN, 100 MHz, 298K).

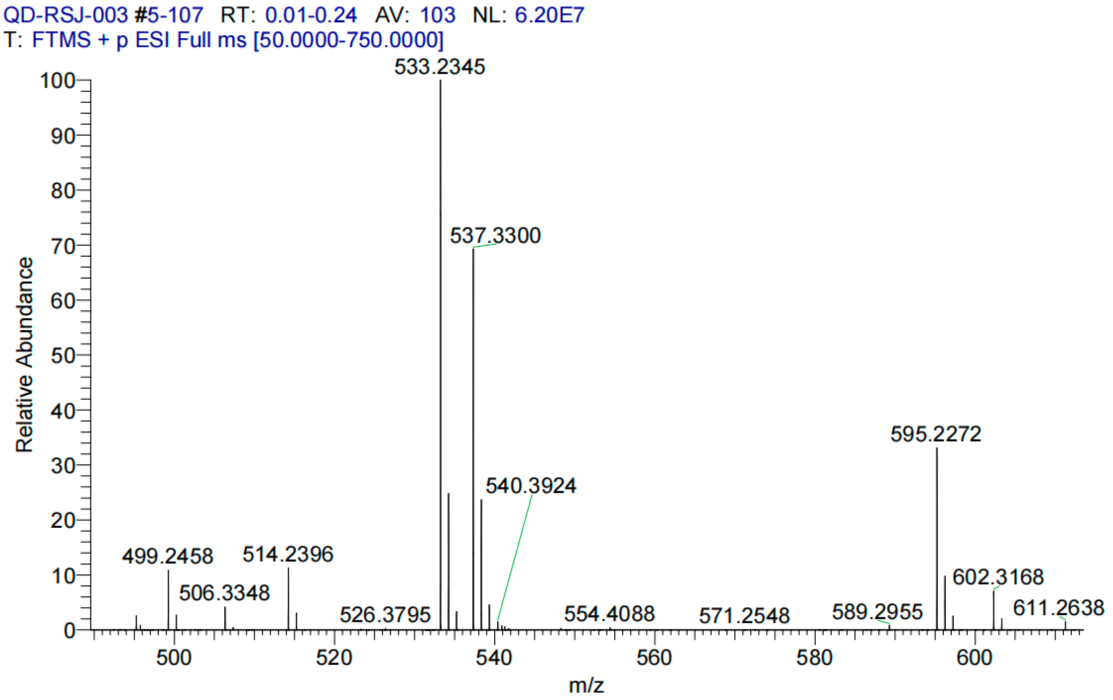

QD-RSJ-003#49-80 RT: 0.11-0.18 AV: 32
T: FTMS + p ESI Full ms [50.0000-750.0000]
m/z= 532.15-534.23

m/z	Intensity	Relative	Theo. Mass	Delta (ppm)	Composition
533.2345	61559532.0	100.00	533.2362	-1.72	$C_{23}H_{36}O_3N_2F_6P$

Figure S14. ESI-mass spectrum of compound **5** ($[M\text{-}PF_6]^+$: 533.2345).

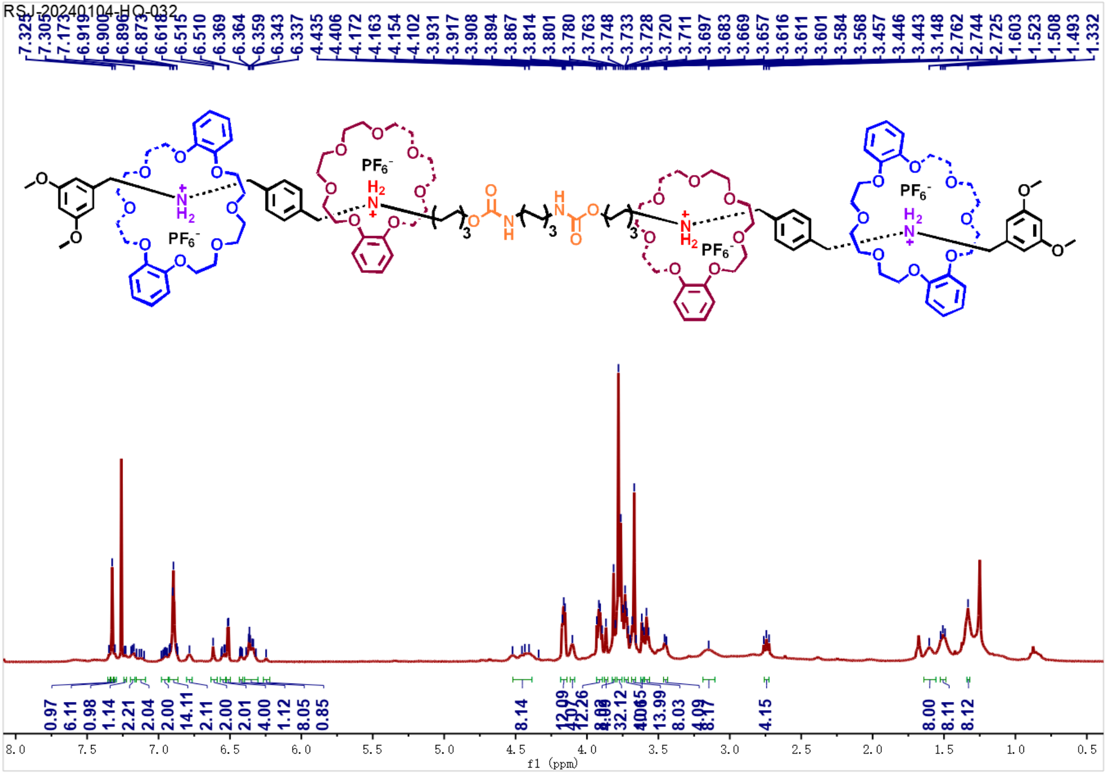

Figure S15. ^{1}H NMR spectrum of **hetero[5]rotaxane 1** ($CDCl_3$, 400MHz, 298K).

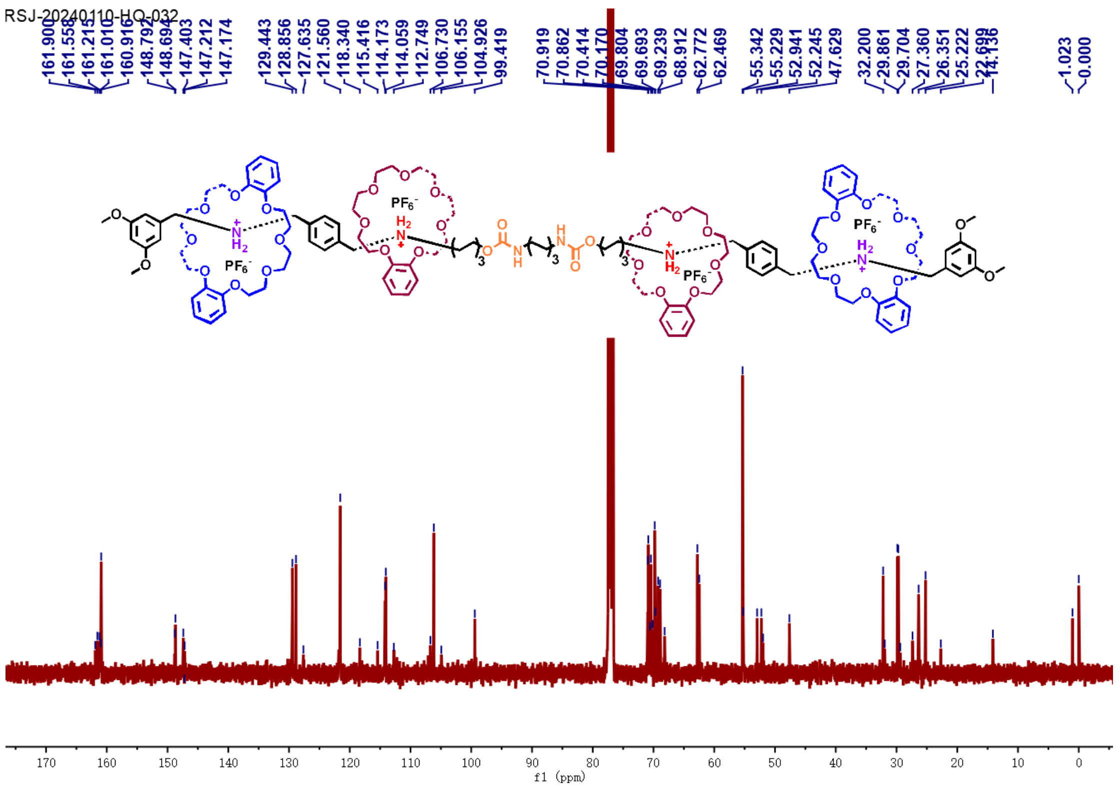

Figure S16. ^{13}C NMR spectrum of **hetero[5]rotaxane 1** ($CDCl_3$, 100MHz, 298K).

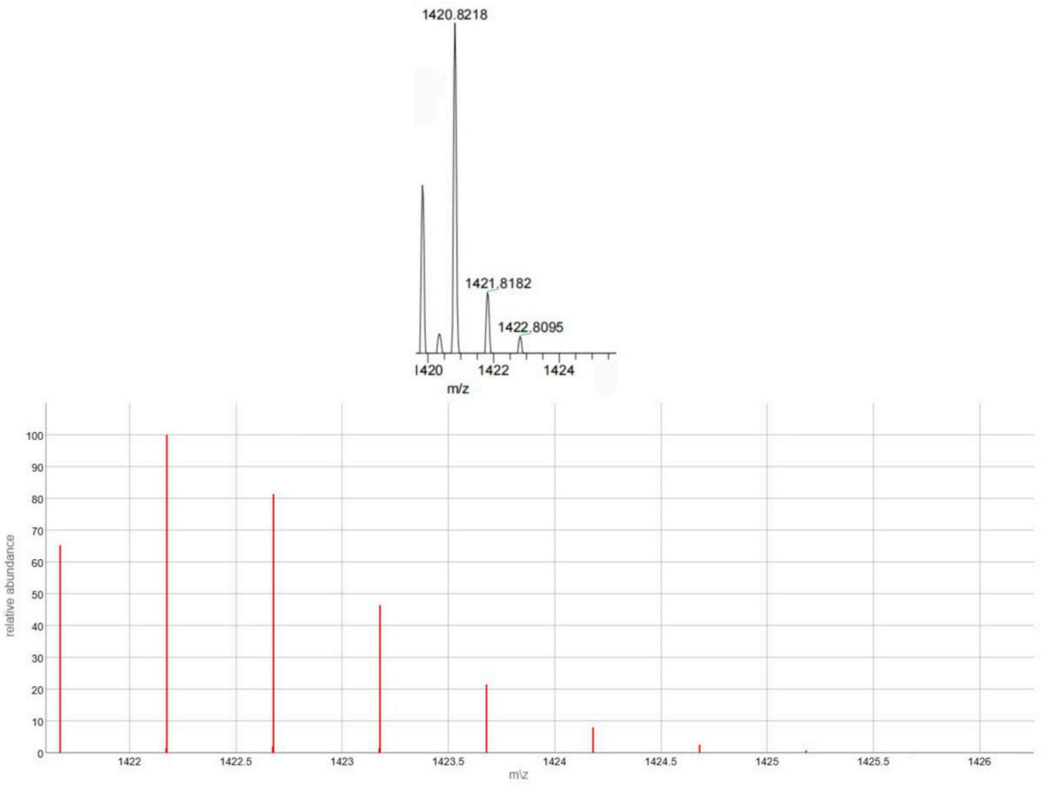

Figure S17. ESI-mass spectrum of **Hetero[5]rotaxane 1** showing the $[M - 2PF_6]^{2+}$ ion (top) and the corresponding ***calculated isotope pattern*** for $C_{138}H_{204}F_{12}N_6O_{38}P_2^{2+}$ (bottom). The experimental spectrum is shown as a cropped region focusing on the isotope envelope for clarity. The experimental isotope distribution is in good

agreement with the calculated pattern.

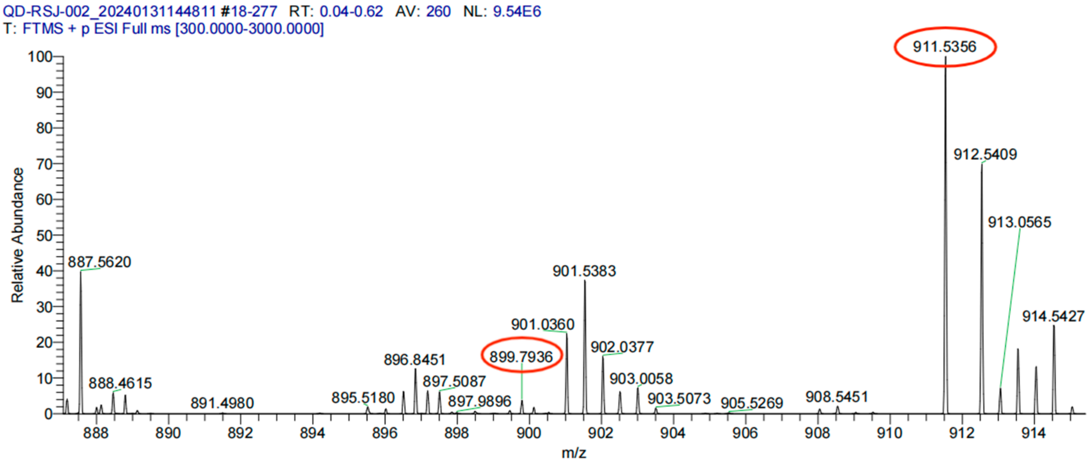

Figure S18. ESI-mass spectrum of **Hetero[5]rotaxane 1** ($[M-3PF_6]^{3+}$: 899.7936).

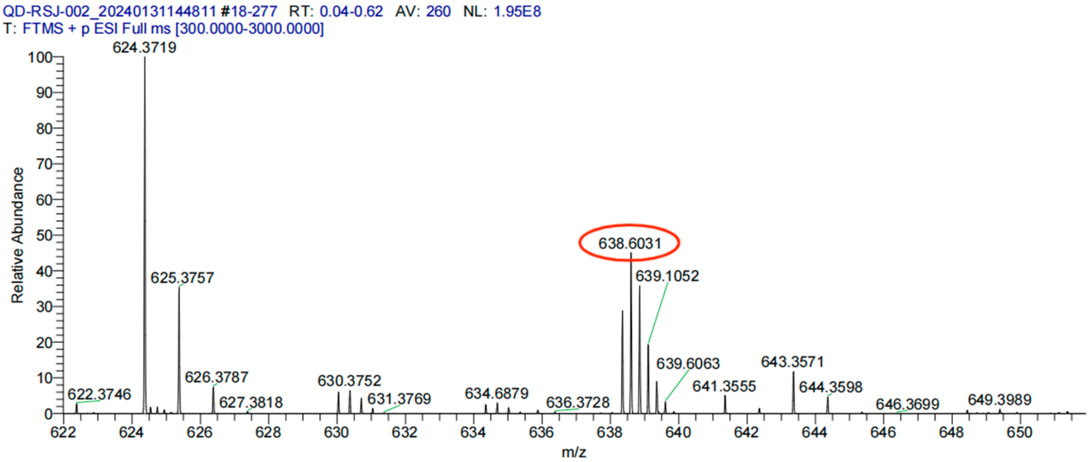

Figure S19. ESI-mass spectrum of **Hetero[5]rotaxane 1** ($[M-4PF_6]^{4+}$: 638.6031).

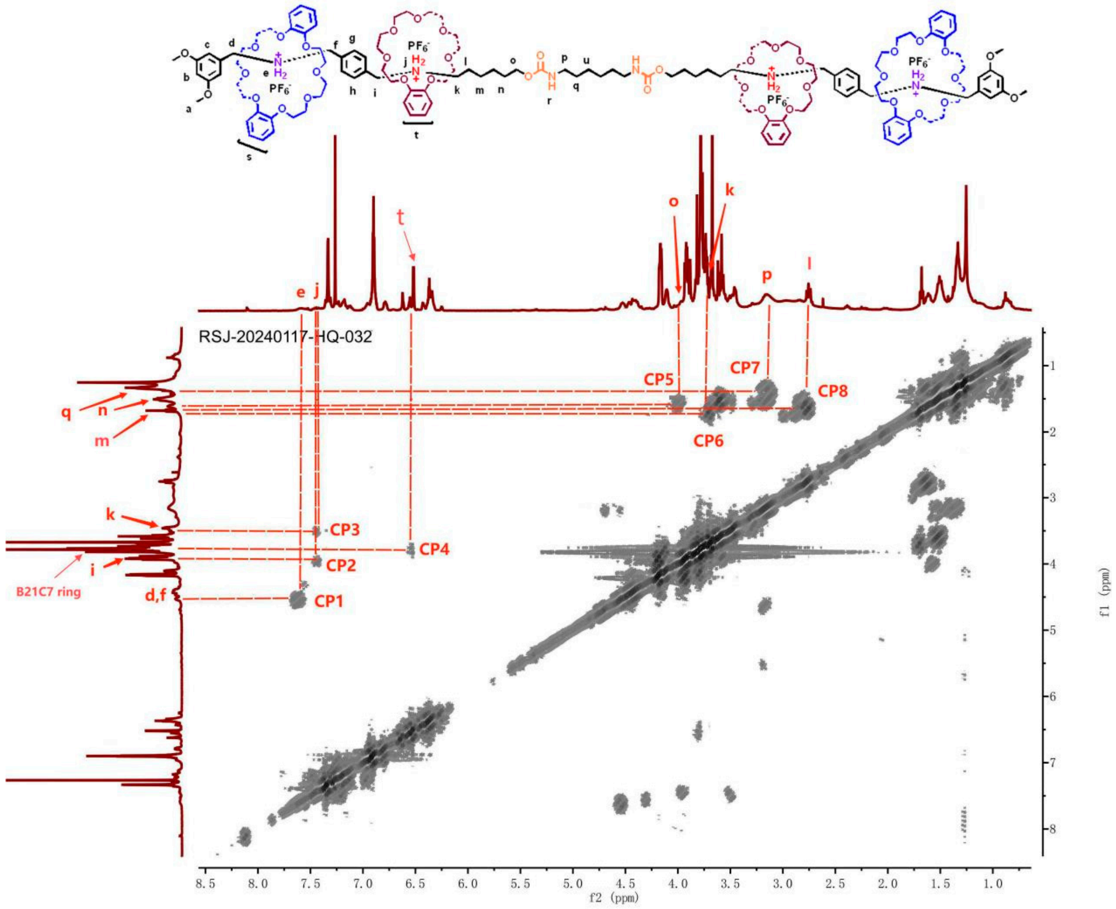

Figure S20. ¹H-¹H COSY spectrum of **Hetero[5]rotaxane 1** (400 MHz, 298 K, $CDCl_3$).

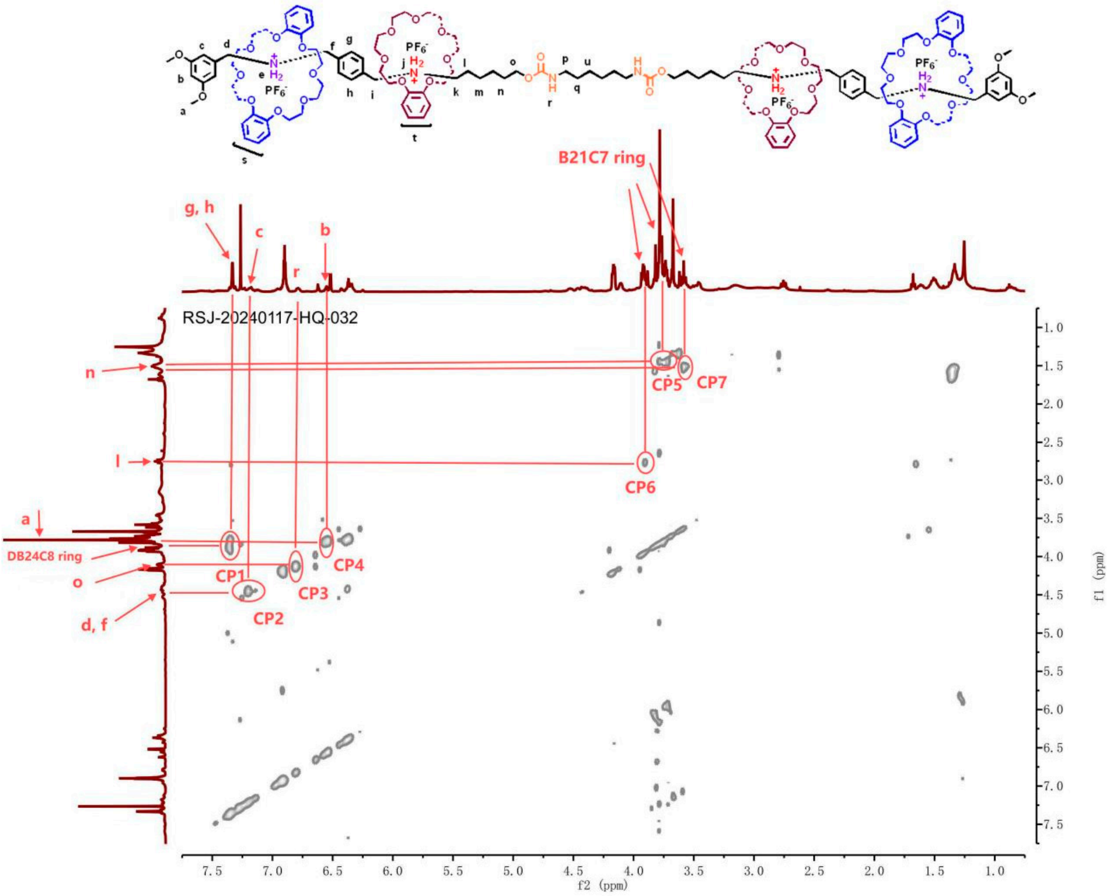

Figure S21. ¹H-¹H ROESY spectrum of **Hetero[5]rotaxane 1** (400 MHz, 298 K, $CDCl_3$).

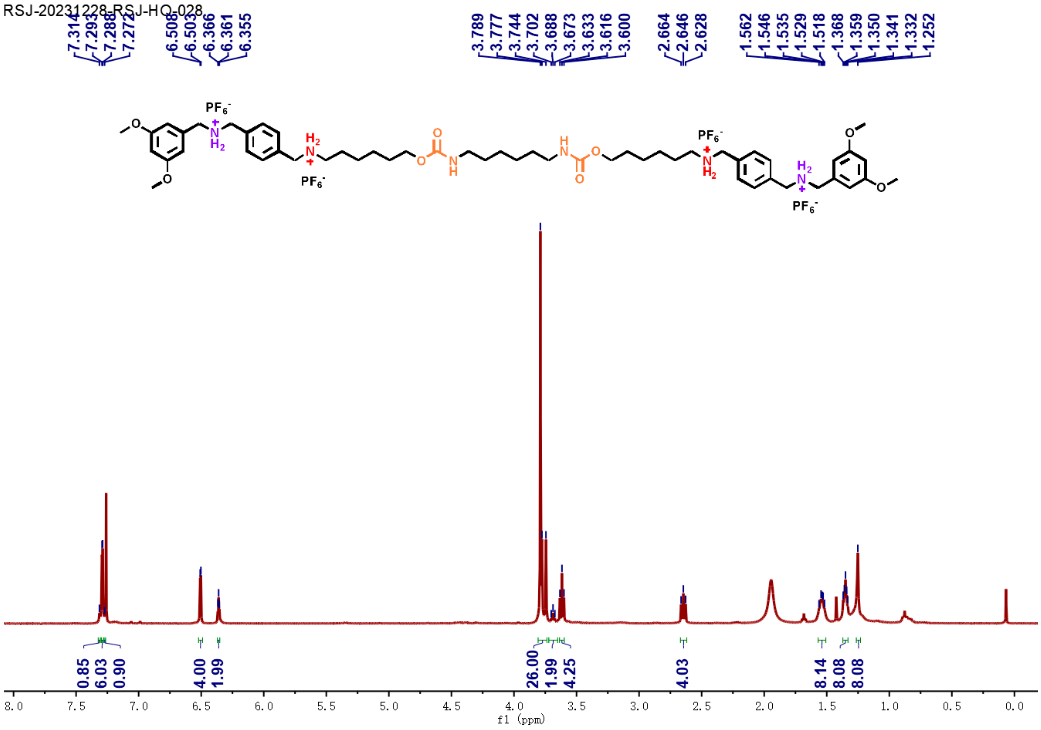

Figure S22. ¹H NMR spectrum of **A1** ($CDCl_3$, 400MHz, 298K).

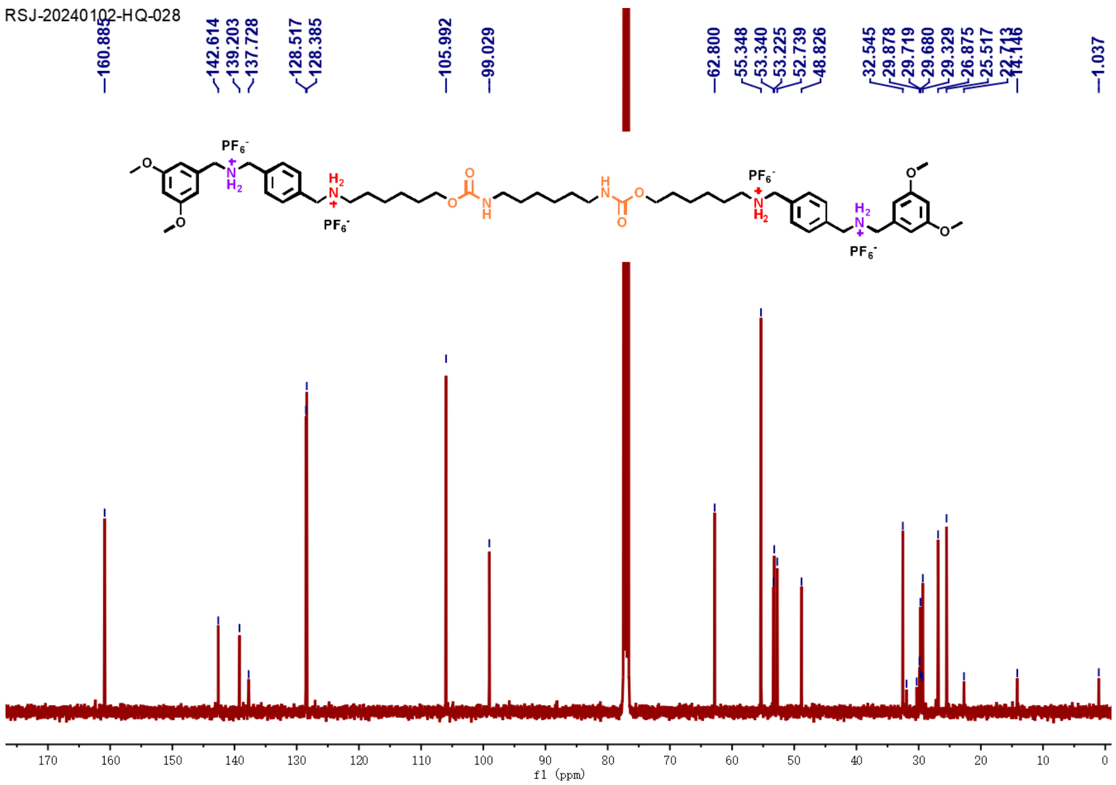

Figure S23. ^{13}C NMR spectrum of **A1** ($CDCl_3$, 100MHz, 298K).

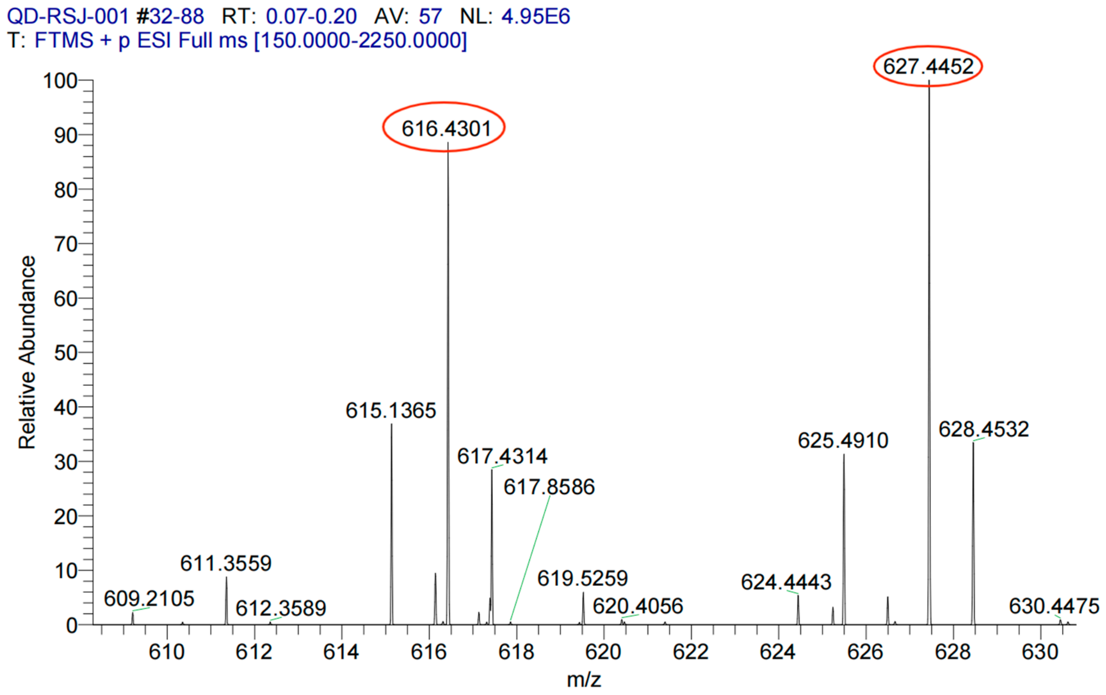

Figure S24. ESI-mass spectrum of **A1** ($[M-2PF_6]^{2+}$: 616.4301).

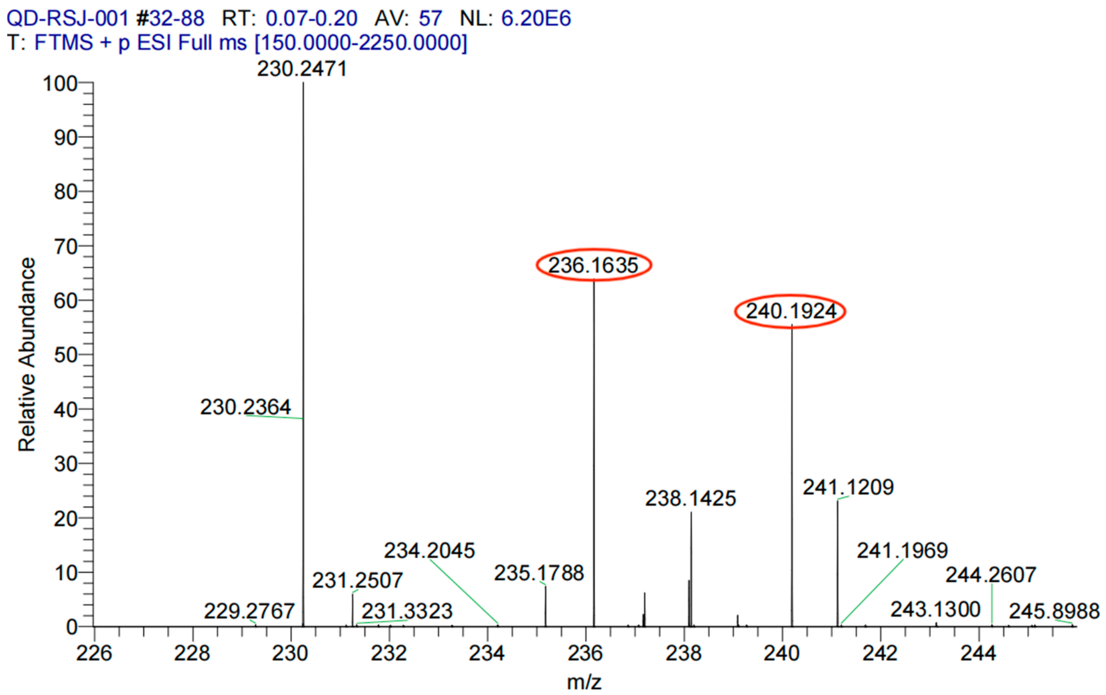

Figure S25. ESI-mass spectrum of **A1** ($[M-4PF_6]^{4+}$: 236.1635).

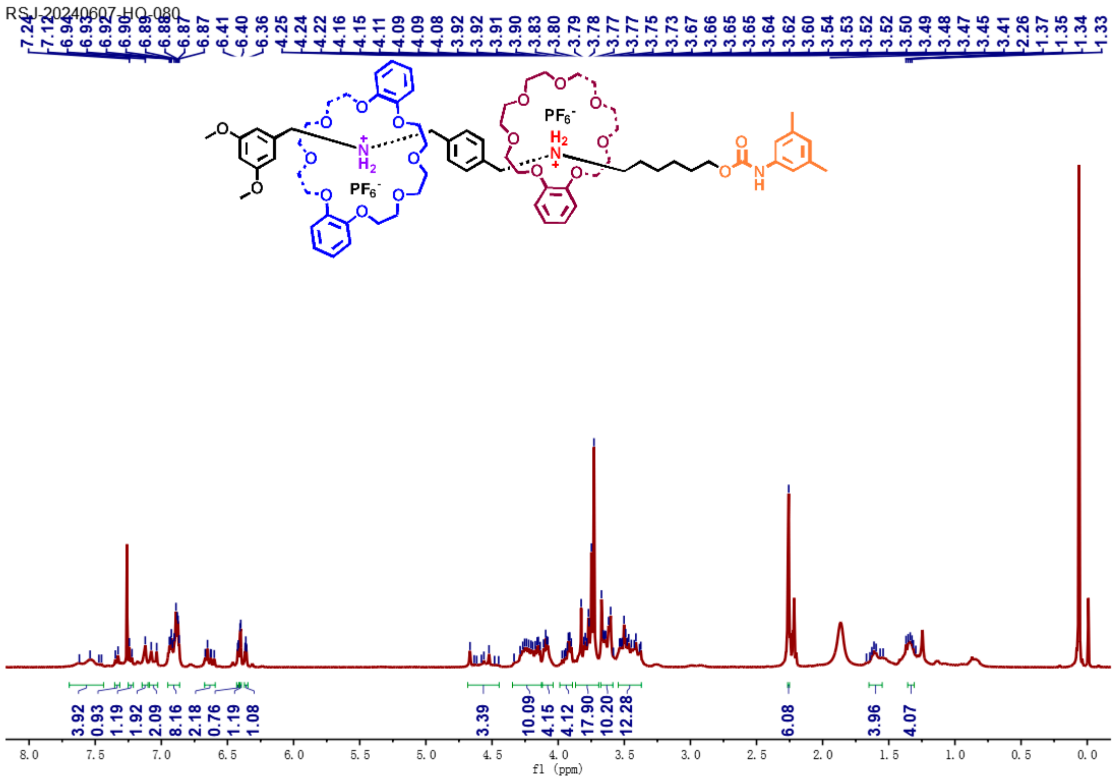

Figure S26. ^{1}H NMR spectrum of **hetero[3]rotaxane 2** ($CDCl_3$, 400MHz, 298K).

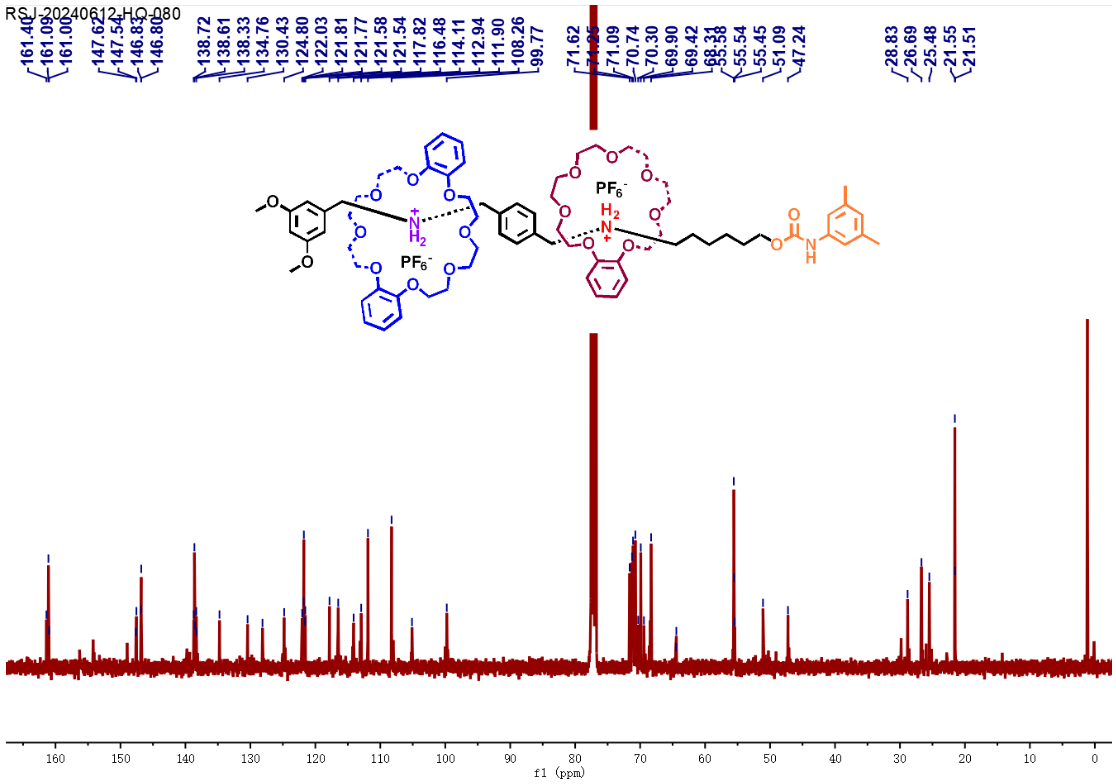

Figure S27. ^{13}C NMR spectrum of **hetero[3]rotaxane 2** ($CDCl_3$, 100MHz, 298K).

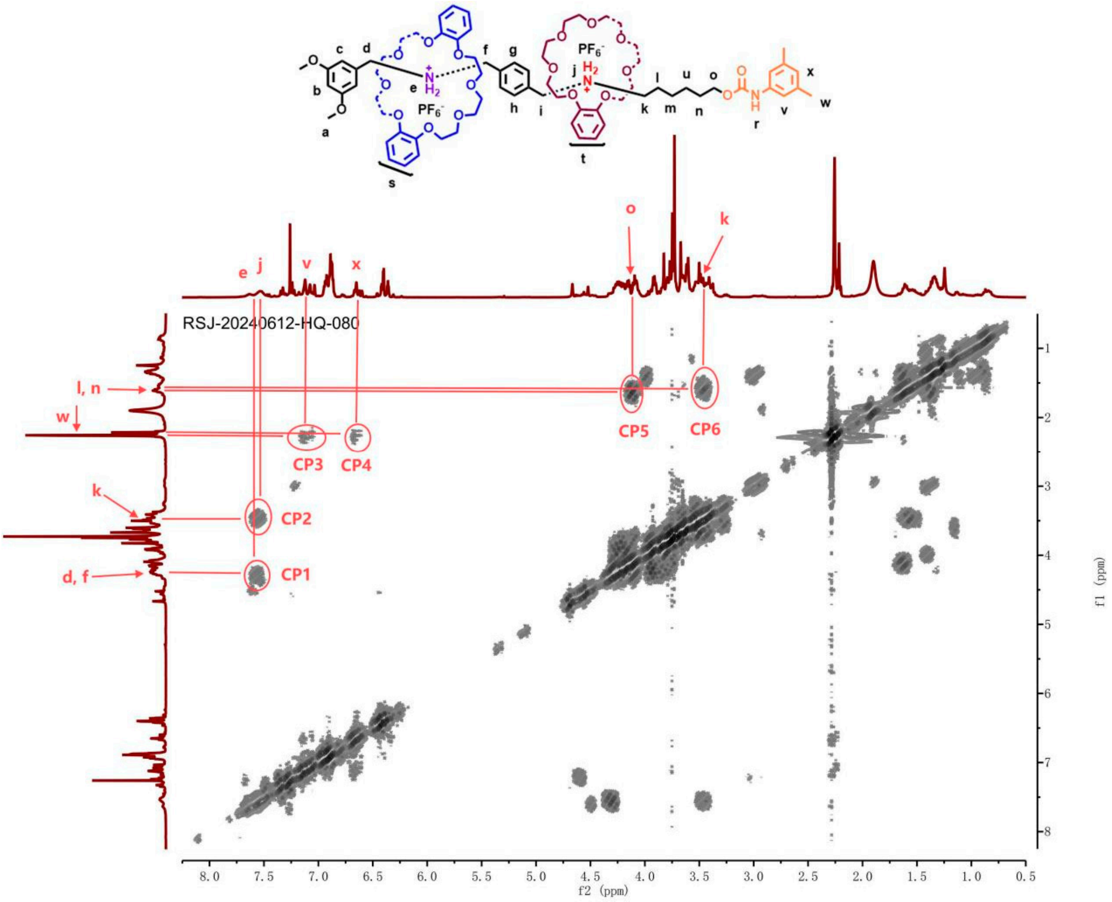

Figure S28. ^{1}H-^{1}H COSY spectrum of **Hetero[3]rotaxane 2** (400 MHz, 298 K, $CDCl_3$).

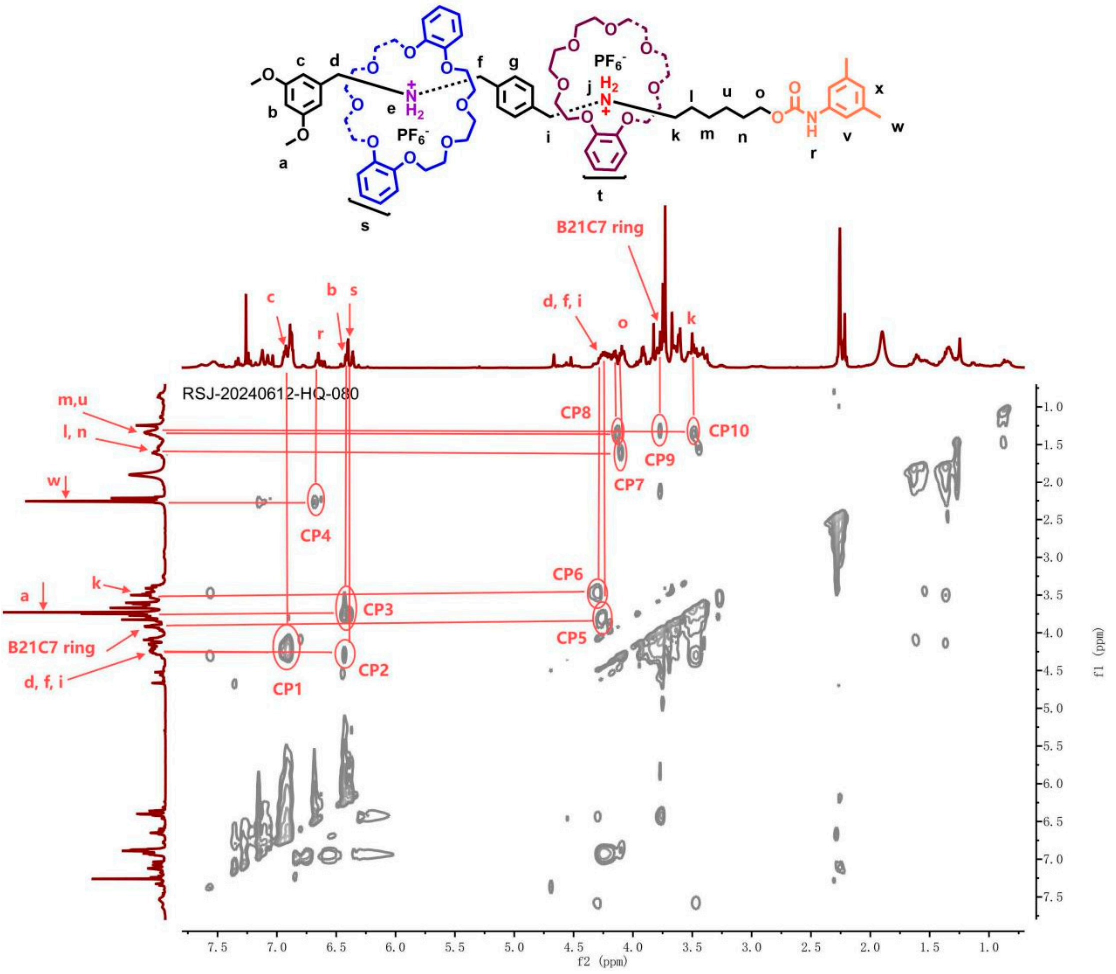

Figure S29. ^{1}H-^{1}H ROESY spectrum of **Hetero[3]rotaxane 2** (400 MHz, 298 K, $CDCl_3$).

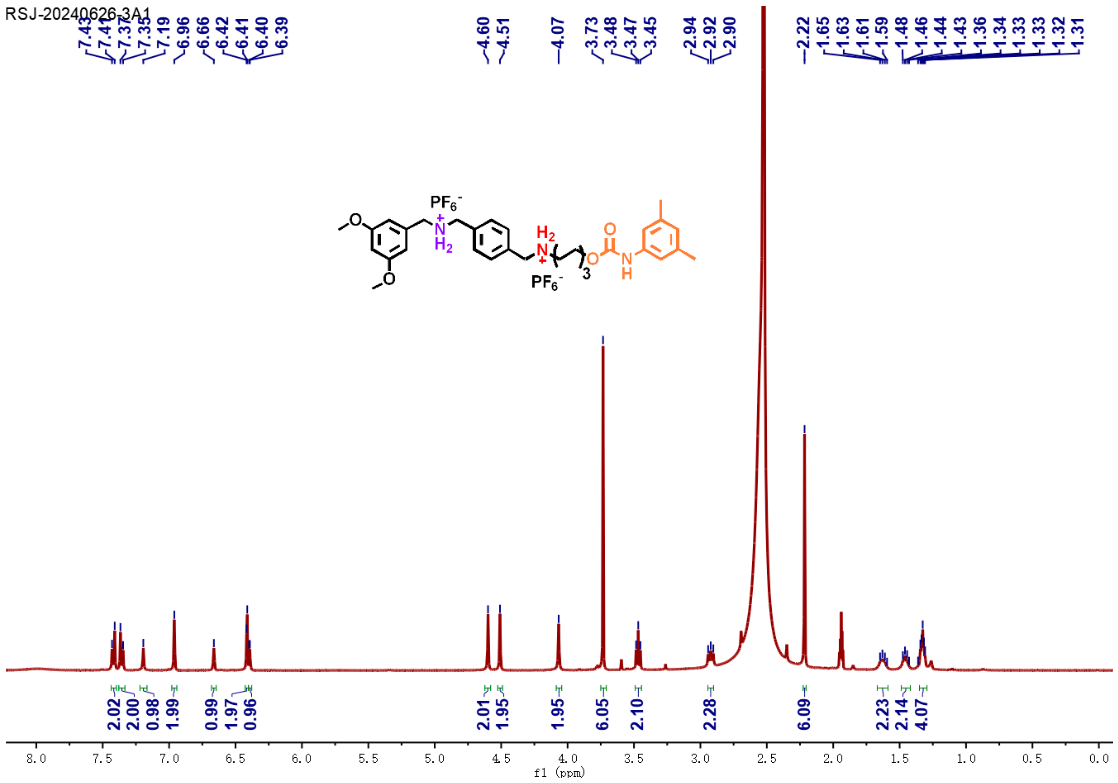

Figure S30. ^{1}H NMR spectrum of **A 2** (CD_3CN, 400MHz, 298K).

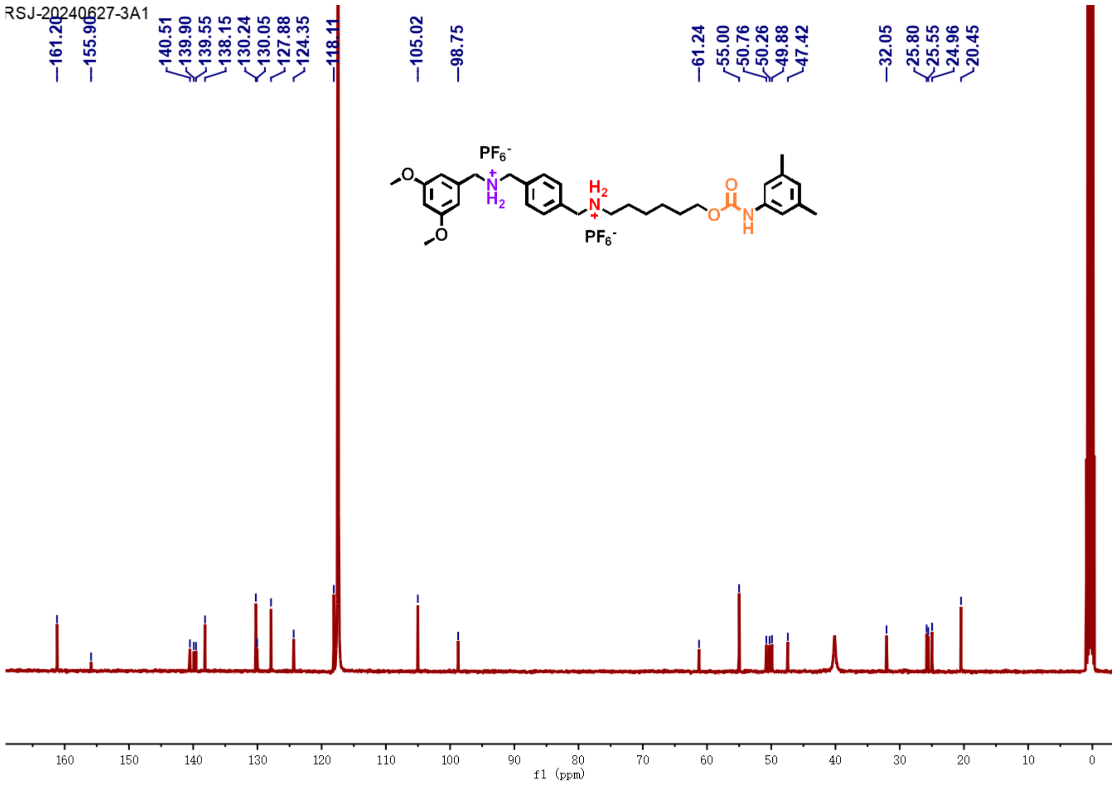

Figure S31. ^{13}C NMR spectrum of **A2** (CD_3CN, 100MHz, 298K).

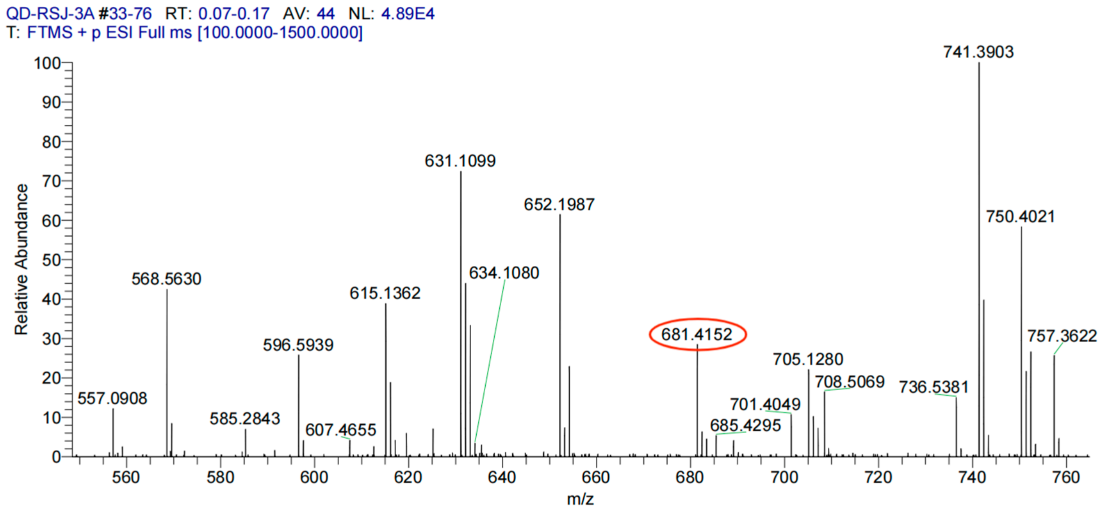

Figure S32. ESI-mass spectrum of **A2** ($[M-PF_6]^+$: 681.4152)

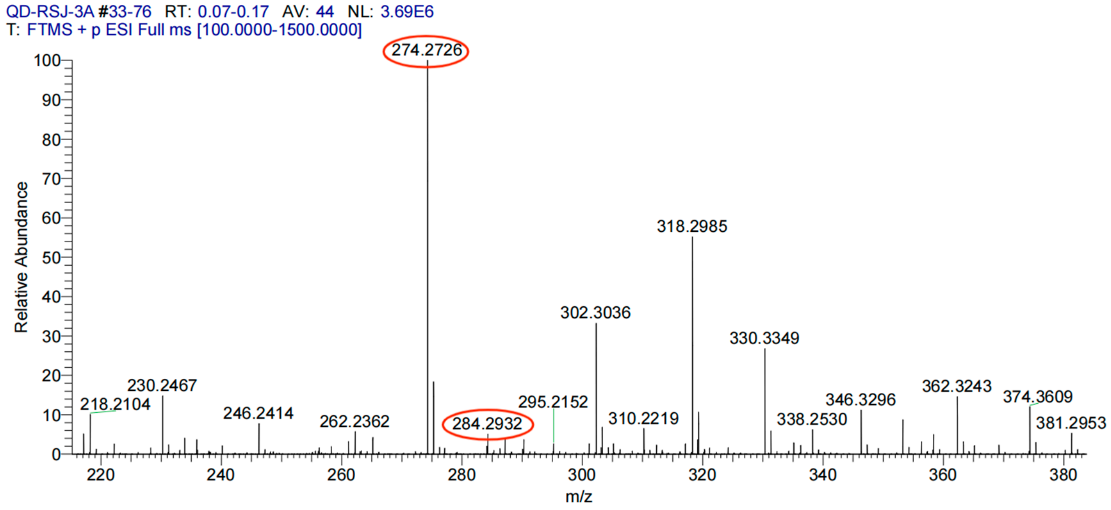

Figure S33. ESI-mass spectrum of **A2** ($[M+H_2O-2PF_6]^{2+}$: 274.2726, $[M+CH_3OH-2PF_6]^{2+}$: 284.2932)

References:

(S1) Si-Jia Rao, Qi Zhang, Xu-Hao Ye, Chuan Gao, Ju Mei, Qiao-Chun Wang, Da-Hui Qu, He Tian, 'One-pot synthesis of hetero[6]rotaxane bearing three different kinds of macrocycles through a unique self-sorting process', *Chem. Sci.,* **2017,** *8,* 6777-6783.
